# Backbone Conformational Equilibrium in Mismatched DNA Correlates with Enzyme Activity

**DOI:** 10.1021/acs.biochem.3c00230

**Published:** 2023-09-12

**Authors:** M. N. Westwood, A. Pilarski, C. Johnson, S. Mamoud, G. A. Meints

**Affiliations:** † Department of Chemistry and Biochemistry, Missouri State University, 901 S. National Ave., Springfield, Missouri 65897, United States; ‡ Biophysics Program, University of Michigan, 930 N. University Avenue, Ann Arbor, Michigan 48109, United States

## Abstract

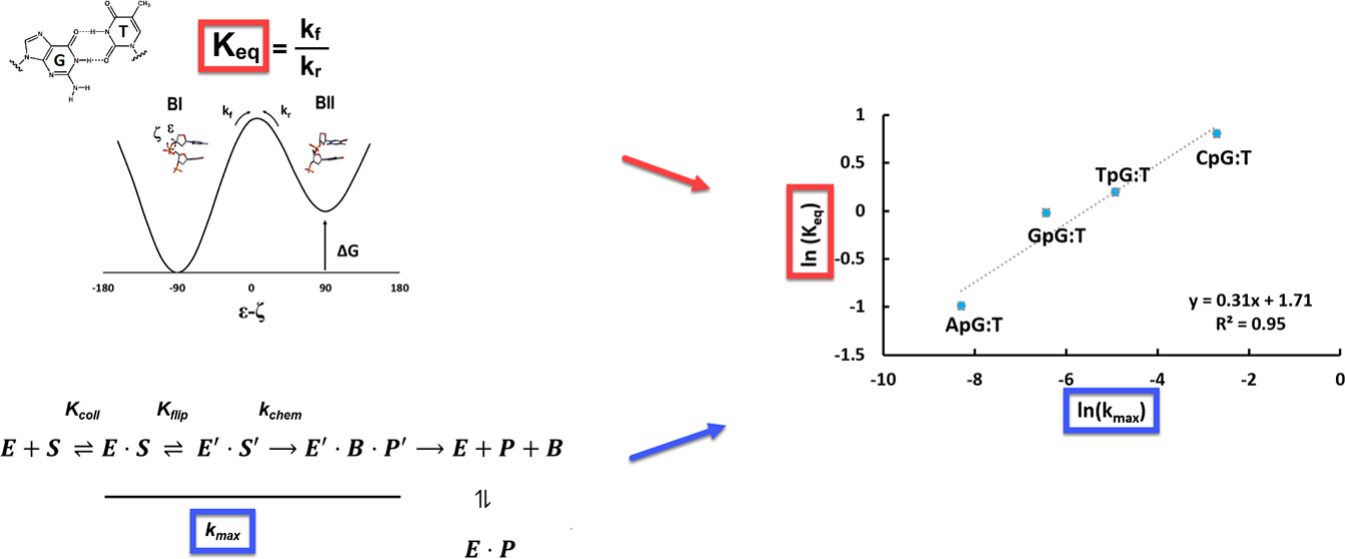

T:G mismatches in mammals arise primarily from the deamination of methylated CpG sites or the incorporation of improper nucleotides. The process by which repair enzymes such as thymine DNA glycosylase (TDG) identify a canonical DNA base in the incorrect pairing context remains a mystery. However, the abundant contacts of the repair enzymes with the DNA backbone suggest a role for protein–phosphate interaction in the recognition and repair processes, where conformational properties may facilitate the proper interactions. We have previously used ^31^P NMR to investigate the energetics of DNA backbone BI–BII interconversion and the effect of a mismatch or lesion compared to canonical DNA and found stepwise differences in Δ*G* of 1–2 kcal/mol greater than equivalent steps in unmodified DNA. We have currently compared our results to substrate dependence for TDG, MBD4, M. **Hha*I*, and CEBPβ, testing for correlations to sequence and base-pair dependence. We found strong correlations of our DNA phosphate backbone equilibrium (*K*_eq_) to different enzyme kinetics or binding parameters of these varied enzymes, suggesting that the backbone equilibrium may play an important role in mismatch recognition and/or conformational rearrangement and energetics during nucleotide flipping or other aspects of enzyme interrogation of the DNA substrate.

## Introduction

Details of recognition and conformational interrogation by DNA-binding proteins remain an important biophysical question. DNA glycosylases manipulate the local structure of DNA (e.g., nucleotide flipping) while searching for and removing aberrant bases during base excision repair (BER),^1−4^ and other enzymes cause an array of structural perturbations.^5^ DNA methyltransferases such as M. **Hha*I* from bacteria^6^ also utilize nucleotide flipping, which has now been observed to be ubiquitous in plants and mammals in methylation, demethylation, and epigenetic oxidation.^7^ There is strong evidence that the scope of the search by DNA glycosylases is reduced by short-range hopping and sliding mechanisms via facilitated diffusion.^8−10^ Nevertheless, the distinguishing local conformational features of a mismatch or lesion that initiate efficient discrimination during facilitated diffusion and leading to nucleotide flipping remain unclear. Mismatches “pre-pay” energetic costs to structural rearrangement during protein binding.^11^ DNA-binding proteins make numerous contacts with the phosphate backbone,^12^ and it has been suggested that “phosphate tracking” is an important aspect of the local search by glycosylases.^10^ From the X-ray structure of M. **Hha*I* with mismatched DNA, it was concluded that the tighter binding indicates a reduction in the energy required to flip a base and that the backbone is the primary target for nucleotide flipping and during the initial stages of flipping the base is merely “carried along,”^13^ while specific base contacts are established during later stages of catalysis. Characterizing the conformational details of the DNA phosphate backbone will provide valuable insight into the recognition and binding processes by a variety of different enzymes.

We have previously observed that T:G mismatches in DNA (as well as U:G mismatches and the lesion 1,N^6^-ethenoadenine) perturb the phosphate backbone conformational equilibrium locally to the mismatch site^14^ by altering the Δ*G* by as much as 1 kcal/mol and the *ΔH* by as much as 4 kcal/mol in the phosphates flanking the mismatch. Mismatches also modify the free energy of activation between backbone conformations Δ*G*^‡^^15^ by as much as 1 kcal/mol and the *ΔH*^‡^ by more than 5 kcal/mol. While there were systematic, *qualitative* trends in these data, there was no *quantitative* evidence comparing to enzyme properties, and therefore it was an open question of what role, if any, these energetic perturbations might play in the recognition or interrogation of mismatches and lesions. Additionally, these results were all in a common 5′-TpG-3′: 5′-CpG-3′ sequence context. But there is no information about whether all T:G mismatches would display this kind of energetic perturbation, or whether there would be a direct connection to enzyme activity. Additionally, there was no overt comparison of the different DNA substrates (i.e., specific base pairs) to enzyme activity.

We currently evaluate the sequence and base-pair dependence of the DNA backbone conformational properties in mismatched DNA for a direct comparison to enzyme activity. T:G mismatches primarily arise in mammals by deamination of 5-methyl-cytosine in CpG steps^16,17^ and by replication errors.^16^ Repair of deamination products is initiated by redundant systems of DNA glycosylases to remove the thymidine. These enzymes include thymine DNA glycosylase (TDG) as well as methyl binding domain glycosylase (MBD4),^18^ which utilize nucleotide flipping during their removal of the target thymine. Extensive biochemical studies^19−24^ have demonstrated a strong dependence of TDG activity on sequence context and base-pairing in the DNA substrates. The sequence dependence is indicated by the identity of the nucleotide 5′ to the base-paired G given by sequence ID (e.g., CpG:T) or alternately by designating the neighbor 3′ to the mismatched T (e.g., G·TG) as both nomenclatures appear in the literature and for completeness are both given in Table 1. The trend in the activity of the TDG enzyme is CpG:T ≫ TpG:T > GpG:T> ApG:T. There is specificity for the second nucleotide as well,^24^ but to a lesser degree than the nearest neighbor. The catalytic domain of MBD4 demonstrates much lower sequence dependence than TDG;^25^ other BER enzymes such as MuTY^26^ and AAG^27^ also demonstrate modest levels of sequence dependence. Methyltransferases such as M. **Hha*I*(28) and DNMT3^29^ display dependence on the cognate binding sequence as well as the flanking sequence, respectively. The enzyme activity on various base-paired substrates (e.g., T:G, U:G, C:G, T:A, etc.) has also been studied extensively^22,23^ and other types of proteins such as CEBPβ have varying degrees of activity and binding strength on DNA containing T:G mismatches.^30,31^

**Table 1:**
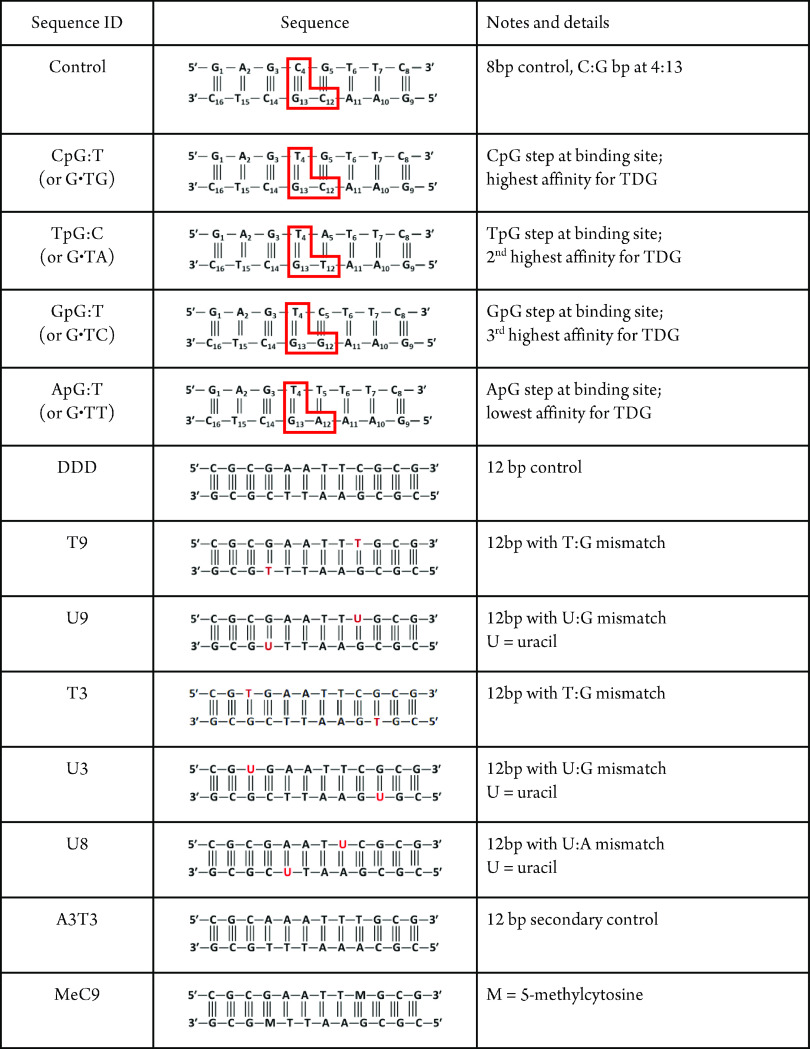
DNA Duplexes Analyzed for This Work^a^

a Note that the non-palindromic sequences have the nucleotide numbering scheme provided and the most relevant nucleotides boxed in red to highlight the sequence dependence.

**Figure 1:**
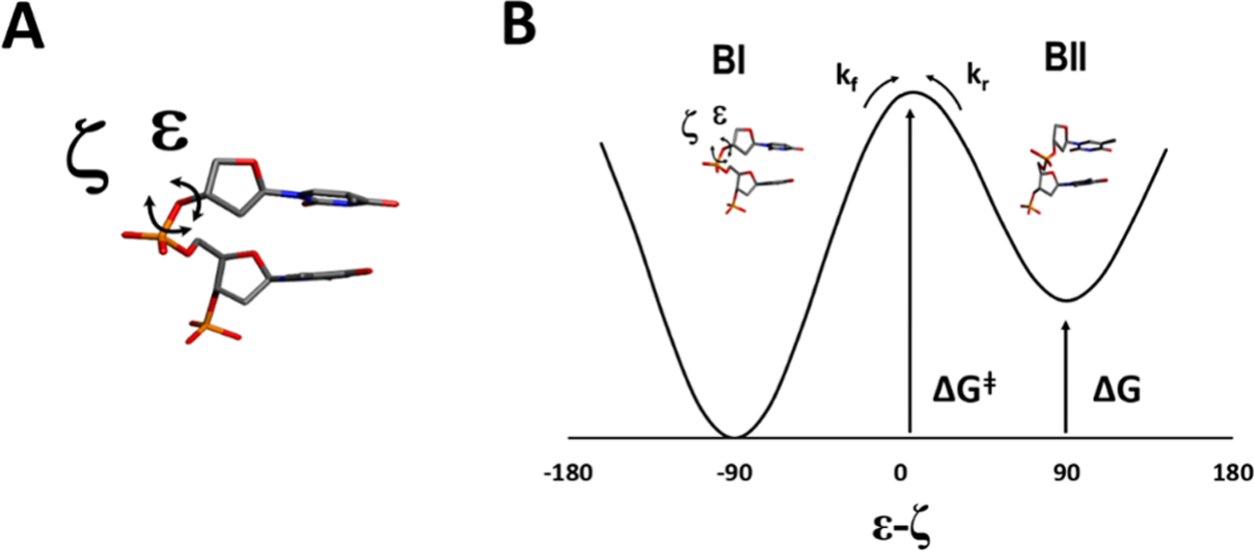
(A) Definition of the backbone dihedral angles most associated with the BI–BII equilibrium (ε and ζ). (B) Illustration of the putative BI–BII interconversion equilibrium energy diagram, the backbone conformations, and the definition of the Gibbs free energies associated with the equilibrium with respect to the forward process.

To attempt to quantify a relationship to enzyme properties, we have determined the equilibrium conformational populations of the DNA backbone using ^31^P solution NMR. The specific conformation of the DNA phosphodiester backbone is described by a series of dihedral angles,^32,33^ where the angles ε and ζ for the primary conformations are traditionally defined (Figure 1) as *trans/g*- in BI (ε - ζ ∼ −90°) and *g-/trans* in BII (ε - ζ ∼ +90°). A large body of evidence has concluded that the DNA backbone is in a dynamic conformational equilibrium between these two primary states BI and BII,^32−35^ modeled as a two-site intramolecular fast exchange on the NMR timescale with unequal forward and reverse rates
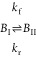
1To quantify the populations of equilibrium states, empirical relationships between the observed ^31^P chemical shifts (δ*P*) and the populations of the two primary conformations (designated as %BI and %BII) have been established.^14,34,35^ These populations directly relate to the Δ*G* and equilibrium constant *K*_eq_ by the standard expression
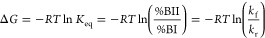
2

The populations of the backbone states BI and BII are directly related to both the temperature and the ^31^P isotropic chemical shift value, which are uniquely identified from 2D NMR as included in the SI. The observed ^31^P isotropic chemical shifts (δ*P*) of the backbone phosphates are therefore the population-weighted average of the isotropic chemical shifts from the two pure states (i.e., Ω_BI_ and Ω_BII_), Ω̅ = *p*_BII_Ω_BII_ + *p*_BI_Ω_BI_ = δ*P*.^14^

We have analyzed five new DNA oligonucleotide duplexes and additionally reevaluated our results from the eight previously published duplexes (Table 1). We assigned their ^31^P isotropic chemical shifts as a function of temperature, which were used to quantify the value of *K*_eq_ and Δ*G* for each phosphate and compared to enzyme kinetic constants. We have examined results from our new duplexes to specifically evaluate the sequence specificity of TDG and used the entire body of our results to compare to base-pairing substrate dependence for TDG, MBD4, M. **Hha*I*, and CEBPβ, for which parameters relating to mismatch binding were available in the literature. We found strong correlations of our DNA phosphate backbone equilibrium (*K*_eq_) to different enzyme kinetics or binding parameters of these varied enzymes, suggesting the backbone equilibrium may play an important role in mismatch recognition and/or conformational rearrangement and energetics during nucleotide flipping or other aspects of enzyme interrogation of the DNA substrate.

## Materials and Methods

Our NMR procedures were used as previously. We analyzed five non-palindromic sequences both to examine sequence specificity and to remove any possible cross-strand effects from self-complementary duplexes. We used 8bp duplexes to reduce spectral overlap in the ^31^P dimension, which remains a significant obstacle even in these short sequences. Throughout the manuscript when “mismatch” is used in the context of our data, we are referring to the T in the T:G base pair, as the G (referred to as the base-pairing partner) is assumed to be the correct nucleotide based upon the C:G context.

### Sample Preparation and NMR Spectroscopy

Custom DNA oligonucleotides were synthesized by IDTDNA (Coralville, IA). Deuterium oxide was purchased from Cambridge Isotope Labs (Tewksbury, MA). All other chemicals were purchased from Sigma-Aldrich (St. Louis, MO). DNA samples were annealed in ∼1.0 mL of 25 mM phosphate buffer (sodium salt), adjusted to pH 7.4 with a dsDNA concentration between 0.5 and 1.0 mM at a final volume of 1.0 mL. Samples were heated in a water bath at 90 °C for 15 min before being allowed to slowly cool for at least three hours. The pH was readjusted with dilute phosphoric acid or sodium hydroxide, and the samples were lyophilized. The dried samples were dissolved in D_2_O, pH-adjusted as necessary, and lyophilized. This procedure was repeated two to three times in high-purity D_2_O to remove exchangeable protons.

NMR experiments were performed using a Varian 400 MHz INOVA NMR spectrometer using VnmrJ 4.2 software, a 5 mm inverse detection double-resonance gradient solution probe, temperature control, and wet 1D solvent suppression, following standard protocols.^36−38^ For chemical shift assignments, spectra were collected at 10 °C and 25 °C. The spectral width for the proton channel was set to 4000 Hz. Unless otherwise noted, NOESY spectra used for assignments were obtained using 64 or 128 *t*1 increments with 256 scans per increment, a mixing time of 200 ms, and a relaxation time of 1 s.

1D ^31^P temperature study experiments were performed with a spectral window of 6000 Hz and a relaxation delay of 1–2 s, and 1024 scans were recorded between 5 and 25 °C for temperature studies with 5 °C increments. The 1D experiments were repeated with a coaxial insert containing 85% phosphoric acid used as an external reference set to 0.00 ppm for all ^31^P peaks. ^1^H-^31^P HSQC experiments were performed with 2048 complex points for the t2 dimension, 256 t1 increments, and a relaxation delay of 1–2 s. The spectral width was 4000 Hz in f2 and 1950 Hz in f1.

NMR experiments for assignment purposes were analyzed using ACD/Spectrus 2017.1.1 software (ACD/Labs, Toronto, Canada). All proton peaks were referenced to the temperature-dependent residual HDO peak.^39^ NOESY data were interpreted by analyzing several distinct regions: the aromatic-H2′/H2″ region, the aromatic-H1′ region, the H1′-H2′/H2″ region, the H2′/H2″-H3′ region, the H1′-H4′ region, and the H2′-H2″ region. As needed, spectra were compared to corresponding regions in the TOCSY, as is the established protocol for distinguishing through-space and through-bond correlations.^36−38^

^31^P isotropic chemical shifts (δ*P*) were assigned (±0.02 ppm) by identifying correlations in ^1^H-^31^P HSQC experiments as a function of the same temperature range as the 1D spectra (i.e., 5–25 °C). The primary correlations were between the phosphate peak for a dinucleotide pair (5′-XpY-3′) and the H4′ from the 3′ nucleotide in the pair. Additional peaks in an HSQC were used as possible, including cross-peaks between the H3′ for the 5′ nucleotide in the same dinucleotide pair and the phosphate peak. Once the individual phosphates were assigned from the 2D experiments, their chemical shift values were monitored and extrapolated for all temperatures from the 1D temperature study (Tables in the SI) and used to determine the temperature dependence of the %BII for each peak in each sequence. Using the equation provided on p. S3 of the SI for ref (14), we determine the population of the BII state (i.e., %BII), and from there, we plug directly into eq 2 to calculate *K*_eq_ and Δ*G*. A compilation of ^1^H and ^31^P chemical shifts, representative NOESY and HSQC spectra, ^31^P temperature studies, and temperature-dependent %BII values are provided in Figures S1–S19 and Tables S1–S11, respectively. As examples, at 298 K, a phosphate peak representing 100% BI would have a value of ca. −1.10 ppm and 100% BII would have a value of ca. +0.35 ppm. A phosphate that samples 50% of each conformation would have a ^31^P chemical shift of −0.40 ppm, exemplified by phosphate C12pG13 in the control sequence (see Figure S4). As previously, from our estimated error of ±0.02 ppm for δ*P*, using standard error propagation, this provides an error in %BII of ±2%, an error of ±0.05 in *K*_eq_, and Δ*G* of ±0.05 kcal/mol.

## Results and Discussion

**Figure 2:**
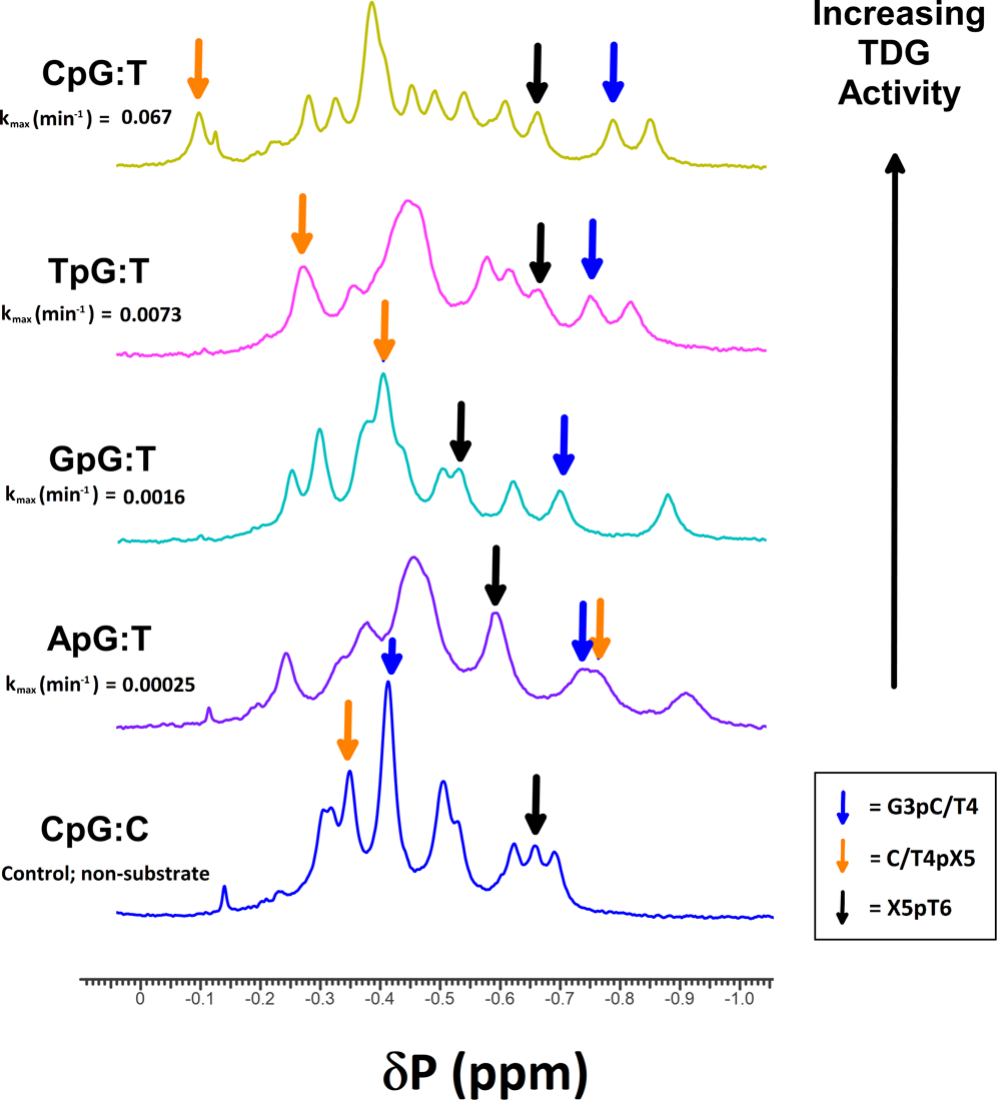
Stacked 1D ^31^P spectra for our five non-palindromic DNA duplexes at 10 °C. Starting at the bottom is our control sequence with a CpG:C context (blue), followed by ApG:T (purple), GpG:T (cyan), TpG:T (pink), and CpG:T (yellow). They are ordered with increasing TDG activity, and their values for *k*_max_ are given.^24^ These spectra have been evaluated for all phosphates, with the three flanking the mismatched T indicated by colored arrows: G3pC/T4 (blue), C/T4pX5 (orange), and X5pT6 (black). Note that the T4pX5 phosphate (orange arrows) in the T:G mismatches has dramatic sequence dependence, whereas the others do not. See Table 1 for complete duplex identity. C/T refers to sequence positions that differ by having a C in a control DNA and a T as a mismatch at the same position.

As discussed, the assignment of the ^31^P isotropic chemical shifts involves first recording a series of temperature-dependent NOESY spectra to assign the nonexchangeable protons. The protons (particularly the H3′ and H4′) correlate with the backbone phosphates, which are subsequently assigned using a series of temperature-dependent ^1^H-^31^P HSQC experiments. From there, we can either use the HSQC or a 1D ^31^P experiment to quantify the backbone equilibrium from the referenced isotropic shifts. Figure 2 compares 1D ^31^P spectra at 283K for the five new non-palindromic sequences described in the sequence dependence study (Table 1) and specifies certain important phosphate peaks and their isotropic shifts in the spectra. As can be seen, the T4pX5 phosphate position (orange, where the T4 is the mismatched T) shows a dramatic dependence upon sequence context.

Based upon this ^31^P NMR data, we calculated the populations of the two primary states BI and BII and equilibrium parameters as previously.^14^ We evaluate %BII, *K*_eq_, and Δ*G* as a function of temperature for all sequences and compare those values relative to a control, unmodified DNA.

**Figure 3:**
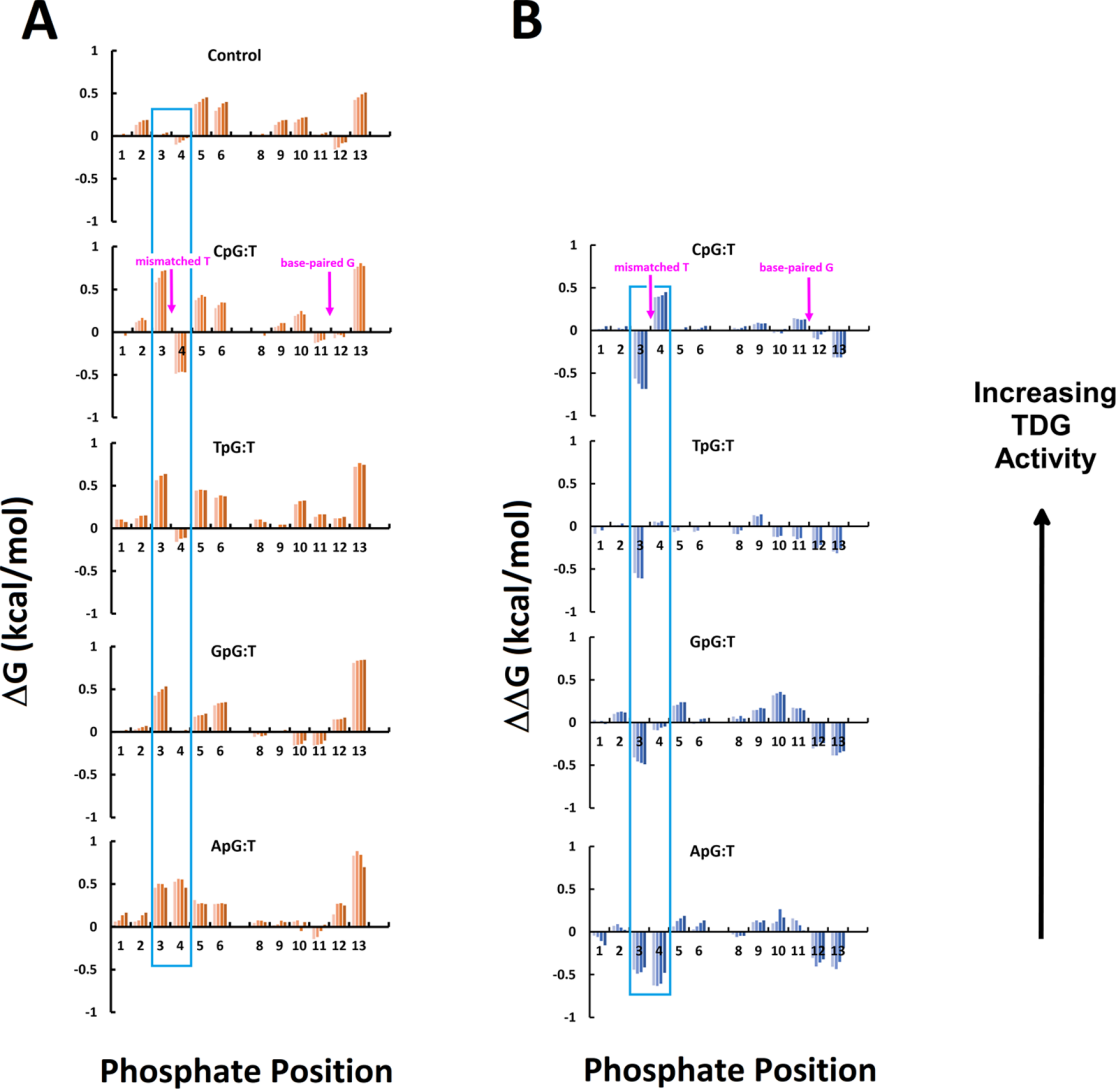
Comparison of Δ*G* (A) and ΔΔ*G* (B, defined as Δ*G*_ctrl_ – Δ*G*_test_) as a function of phosphate position (horizontal axis), temperature (bars) from 5 to 20 °C (except TpG:T, where the 20 °C experiment gave poor data), and sequence dependence. The mismatched T and base-paired G are indicated by arrows. The boxes highlight the phosphate positions flanking the mismatched T, demonstrating the significant sequence dependence. Note that the 3′ ends are eliminated due to melting issues at the higher temperatures. Uncertainty in Δ*G* values was determined to be ±0.01 kcal/mol based upon standard error propagation from 0.02 ppm uncertainty in ^31^P isotropic chemical shift values.

The results are exemplified in Figure 3, which shows the relative difference in Δ*G* for a mismatched sequence to a control sequence, or ΔΔ*G*. There is a large stepwise difference in Δ*G* for the two phosphates flanking the mismatched T in the sequence with the CpG:T sequence context. In other words, there is a step from a phosphate with a relatively high population of BI to a step with a relatively high population of BII on the phosphates that flank (5′ and 3′, respectively), the mismatched base pair. This trend is equivalent to what we have observed previously^14,15^ and is not present in the control sequence (containing a C:G base pair). The results are interesting when evaluated in mismatched sequences with different flanking bases (5′ to the base-paired G) substituted from C → T → G → A. There is a dramatic reduction in the population of BII, with a corresponding decrease in *K*_eq_ and an increase in +Δ*G* on the 3′ side of the mismatched T (Figure 3) following this order of samples.

**Figure 4:**
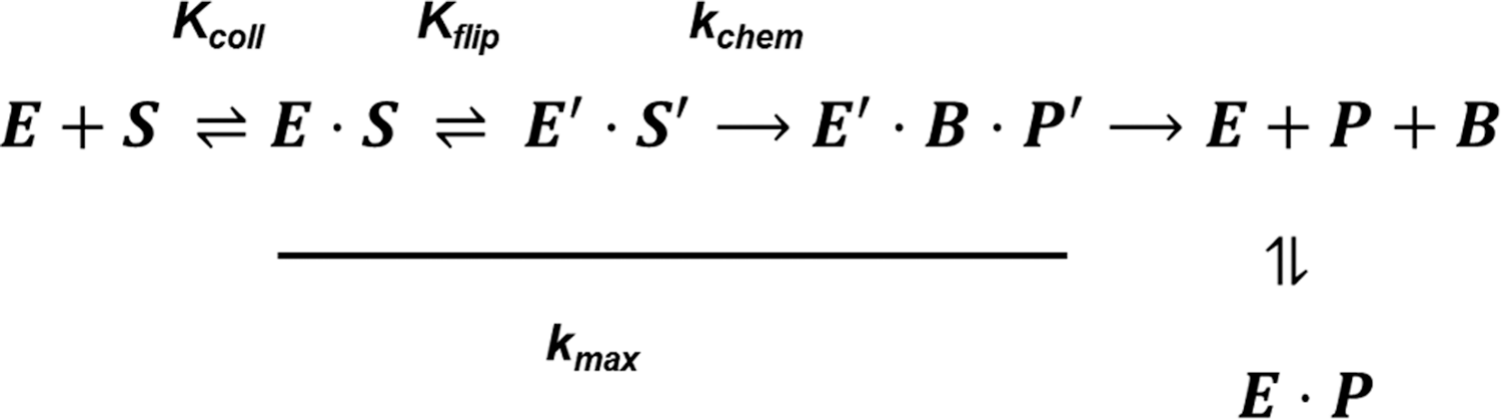
Minimal kinetic mechanism as adapted from Dow et al.^24^ Copyright 2019 American Chemical Society.

It is important here to discuss the proposed mechanism of TDG in as much detail as possible, within the context of our results. Figure 4 shows the minimal kinetic mechanism as proposed by Dow et al.^24^ It is a multistep process highlighted by initial binding equilibrium (*K*_coll_), followed by a nucleotide flipping equilibrium (*K*_flip_), then the chemistry steps (*k*_chem_ and/or *k*_max_).

A qualitative relationship between the DNA backbone and mismatch sequence context was initially observed^40^ in the context of the Mismatch Repair (MMR) rather than Base Excision Repair. A large chemical shift perturbation in the ^31^P chemical shift was observed in a CpG:T context that was not observed in an ApG:T context and extrapolated to assume a larger value of BII at that site. However, it was concluded that any relationship between the DNA backbone and mismatch recognition by human homologues to the MutS repair proteins was unlikely. Our results in CpG:T contexts suggest that what occurs is a dramatic conformational shift to BI on the 5′ side of the mismatched T, but on the 3′ side there is a conformational shift to BII, which decreases as a function of flanking nucleotide.

**Figure 5:**
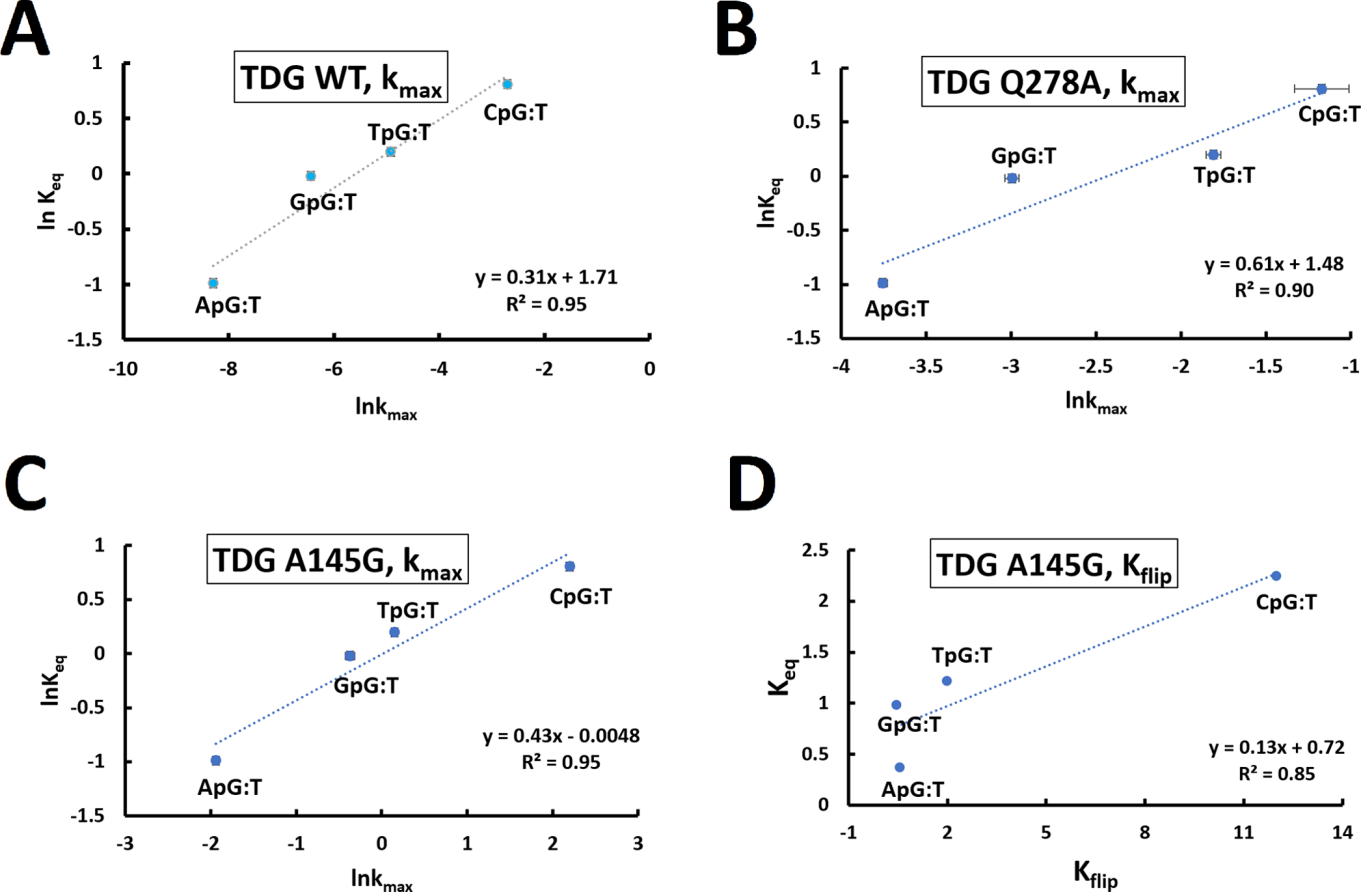
Correlations between DNA backbone equilibrium (ln* K*_eq_ or *K*_eq_) for the T4pX5 phosphate and TDG activity as a function of sequence contexts. (A) WT TDG, (B) Q278A TDG mutant, (C) A145G TDG mutant, (D) A145G TDG mutant comparing *K*_eq_ to K_flip_. All data points have error bars representing the standard deviation of both the NMR data plus the data provided in the original manuscripts. All TDG data adapted from ref (24). Copyright 2019 American Chemical Society. Note the different axes arise due to the different systems and properties being analyzed.

The trend in backbone equilibrium (*K*_eq_) at the phosphate immediately 3′ to the mismatched T nucleotide follows TDG activity (*k*_max_) directly from the highest to lowest activity for each neighboring nucleotide. This is exhibited more quantitatively in Figure 5A, where we plot ln *K*_eq_ vs ln* k*_max_, where *K*_eq_ is defined in Figure 1 and *k*_max_ is defined in Figure 4. Values for *K*_eq_ and all enzyme properties are given in Tables 2 and 3. We evaluated every phosphate (except those on the ends of the duplex) in the set of eight base-pair duplexes (Table 1) for a total of 12 phosphates per sequence and examined correlations with every position. Of all of the phosphates, only the middle four on the mismatched T-containing strand (G3pT4, T4pX5, X5pT6, and T6pT7) had correlations above 0.25. Of those, the T4pX5 phosphate (the phosphate also with the highest correlation) was the only position with a sufficiently large range of values of the ^31^P chemical shift and therefore *K*_eq_. In other words, other positions correlated (e.g., G3pT4), but the range of values of the ^31^P chemical shift and *K*_eq_ in the four sequences was so small as to be irrelevant. Given that there are only four possible arrangements for the base immediately to the 5′ side of the base-paired G, this fundamentally limits the statistics of the analysis and one must be careful about overinterpretation of a correlation with only four points. These results also only reflect comparisons when the first nearest neighbor is changed. There is evidence of a slightly reduced impact of the second nearest neighbor both on the enzyme kinetics^24^ as well as the backbone equilibrium,^15^ but the analysis herein focuses only on the effects of nearest neighbors. Aggregation and normalization of all data contained herein and plotting a comprehensive correlation graph would in principle offer superior statistics. However, given the differences in conditions, such as temperature, DNA length, DNA sequence, enzyme, and enzyme length, as well as the fact that many studies were performed by different groups, it seemed more reasonable to narrow the analysis to leave out as many variables as possible.

**Table 2:** Numerical Values of Conformational Equilibrium Constants and Enzyme Kinetic Parameters Used in Correlations^a^

sequence ID	*K*_eq_ T4pX5 step	*k*_max_ (min^–1^) (TDG WT)^b^	*k*_obs_ (min^–1^) (MBD4)^c^	*K*_flip_ (TDG A145G)^b^^,^^d^	*k*_max_ (min^–1^) (TDG Q278A)^b^	*k*_max_ (min^–1^) (TDG A145G)^b^
control	1.09 ± 0.05					
CpG:T (or G·TG)	2.24 ± 0.05	0.067 ± 0.002	0.1492 ± 0.0080	12	0.31 ± 0.05	9.0 ± 0.3
TpG:C (or G·TA)	1.22 ± 0.05	0.0073 ± 0.0004	0.0993 ± 0.0065	2	0.164 ± 0.007	1.16 ± 0.04
GpG:T (or G·TC)	0.980 ± 0.05	0.0016 ± 0.0001	0.0918 ± 0.0034	0.46	0.050 ± 0.002	0.69 ± 0.04
ApG:T (or G·TT)	0.372 ± 0.05	0.00025 ± 0.00001	0.0555 ± 0.0075	0.57	0.0234 ± 0.0006	0.144 ± 0.05

a Specifically this table compares the sequence dependence of the backbone equilibrium with TDG and MBD4 enzyme activity.

b Adapted with permission from ref (24). Copyright 2019 American Chemical Society.

c Adapted with permission from ref (25). Copyright 2012 Elsevier.

d Error not provided but estimated from data to be ± 0.1 or less. Error in *K*_eq_ is 0.05, by standard error propagation from the ± 0.02 ppm uncertainty in the ^31^P chemical shifts. Enzyme data acquired at 18 °C, and NMR data acquired at 15 °C.

**Table 3:** Numerical Values of Conformational Equilibrium Constants and Enzyme Kinetic Parameters Used in Correlations^a^

comparison of kinetic and thermodynamic parameters				
base pair	*K*_eq_ (25 °C)^b^	*k*_max_ (min^–1^) (TDG, 18 °C)^c^	R (M. **Hha*I*)^d^	*K*_D_ (CEBPβ)^e^
G:C (ave)	1.27 ± 0.05	0.000012 ± 0.00005	0.4 ± 0.08	2.0 ± 0.5
G:T (ave)	3.34 ± 0.05	0.22 ± 0.04	8.25 ± 0.08	0.030 ± 0.005
G:U (ave)	7.34 ± 0.05	2.6 ± 0.3	9.75 ± 0.08	0.06 ± 0.01
A:U	0.477 ± 0.05	0.000053 ± 0.000001		
T:A & A:T (ave)	0.450 ± 0.05	0.000013 ± 0.000003		13^f^
G:M	1.57 ± 0.05		0.0075 ± 0.08	0.7 ± 0.1
A:T	0.486 ± 0.05		0.00625 ± 0.08	
T:A	0.422 ± 0.05		0.015 ± 0.08	

a Specifically this table compares the base pair dependence of the backbone equilibrium with TDG, M. **Hha*I*, and CEBPβ enzyme activity.

b Adapted with permission from ref (14). Copyright 2012 American Chemical Society.

c Adapted with permission from refs (22) and (23). Copyright 2006 American Chemical Society and CC BY 4.0, respectively.

d Adapted with permission from ref (43). Copyright 1995 Oxford University Press.

e Adapted with permission from ref (30). Copyright 2012 Oxford University Press.

f There is no error provided, and the value is given as the lower limit, but we used it directly in our analysis. Error in *K*_eq_ is 0.05, by standard error propagation from the ±0.02 ppm uncertainty in the ^31^P chemical shifts. All NMR data acquired at 25 °C, and enzyme temperature data provided in references.

Ideally, all NMR and enzyme data would be collected at the same temperature and under physiological conditions. However, the sequence-dependent enzyme work was performed at 18 °C,^24^ and we conducted the NMR experiments at 5, 10, 15, and 20 °C. The small oligo length coupled with the mismatch presented challenges due to sample melting even noticeable at 20–25 °C. With all of these considerations, we felt the comparison of the 18 °C enzyme data and the 15 °C NMR data was most relevant and compelling.

Dow et al. investigated the effect of specific site-directed mutants on TDG, specifically Q278A and A145G. The Q278A mutant demonstrated reduced overall activity while the A145G had increased overall activity. However, neither mutation had a significant effect on the sequence specificity, as shown in Figure 5B,C. The authors even state their findings suggest that TDG specificity to the CpG context is due to factors other than interactions between specific amino acid residues and the DNA bases and that the base on the 3′ side of the target T might affect the flipping process. Our results support both of those conclusions.

The enzymatic rate constant *k*_max_ is perhaps not an ideal parameter for comparison, as based on the proposed mechanism it contains multistep information including the chemistry step (Figure 3) which may not be directly related to or dependent on the backbone conformation. A better direct comparison would be *K*_flip_, the constant for the flipped/unflipped equilibrium after TDG binding (Figure 4). For wild-type TDG a full sequence context study of nearest-neighbor nucleotides is unavailable (three values were measured; see ref (24)); nevertheless, comparisons indicate there is a strong correlation (data not shown). There is a full data set for *K*_flip_ with the four nearest-neighbor contexts for the A145G TDG mutant rather than for wild-type TDG. This data is presented in Figure 5D and does demonstrate a good correlation of 0.85.

**Figure 6:**
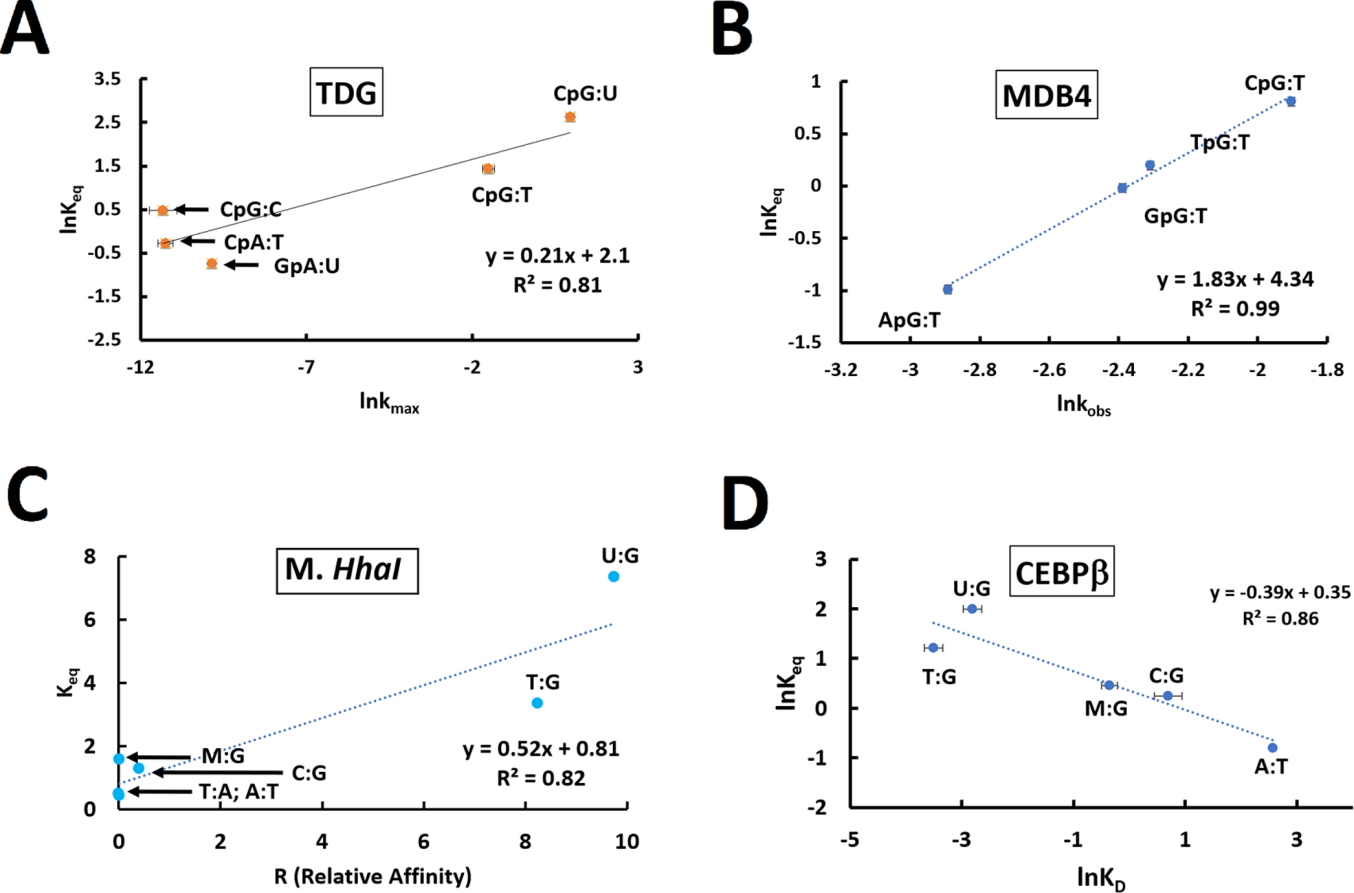
Correlations between DNA backbone equilibrium (*K*_eq_ or ln* K*_eq_) and enzyme activities as a function of base-pairing context. (A) WT TDG *k*_max_. Adapted with permission from refs (22) and (23). Copyright 2006 American Chemical Society and CC BY 4.0, respectively. (B) MBD4 k_obs_. Adapted with permission from ref (25). Copyright 2012 Elsevier (C) M. *Hha*I relative affinity R. Adapted with permission from ref (43). Copyright 1995 Oxford University Press. (D) CEBPβ binding constant (*K*_D_). Adapted with permission from ref (30). Copyright 2021 Oxford University Press. All data points have error bars representing the standard deviation of both the NMR data plus the data provided in the original manuscripts. Note that M:G refers to a 5-methyl-cytosine:guanine base pair. Note the different axes arise due to the different systems and properties being analyzed.

Sequence context is not the only relevant comparison between the enzyme kinetics of TDG and the backbone equilibrium. Analysis of different base substrates and base-pairing contexts is also relevant. We have therefore compared average backbone equilibria (using all sequences analyzed to generate average *K*_eq_ values) for T:G; U:G, U:A, C:G, and T:A base pairs to *k*_max_.^22,23^ The correlation is shown in Figure 6A.

Other DNA-binding enzymes have mismatches as substrates. While human glycosylases TDG and MBD4 both recognize T:G mismatches, it is interesting to note that MBD4 does not have a strong dependence on the flanking sequence the way TDG does.^25^ Nevertheless there is an equivalent strong correlation between its enzyme kinetics (*k*_obs_) and the backbone equilibrium (Figure 6B). TDG, MBD4, and M. **Hha*I* all use nucleotide flipping to access the target base but are otherwise unrelated and serve different primary roles in cellular function. TDG and MDB4 have similar functionality in removing T:G mismatches even though they have almost no amino acid sequence similarity^18^ and are proposed to play distinct but important roles in the epigenetics of cytosine methylation.^17^ Interestingly, MBD4 has both significant homology and structural similarity to repair enzymes MutY and MIG.^25^ Therefore, is it entirely possible that, even though they also share the nucleotide flipping motif and primary substrate, they have different modes of binding and/or recognition of their targets and their different origins. Indeed, even related repair enzymes with similar targets AAG and AlkA appear to have different mechanisms of binding.^41^

Yang et al. directly measured enzyme kinetics for M. **Hha*I* methyltransferase binding to T:G and U:G mismatches^42^ and found a ∼5- and ∼10-fold higher relative affinity than the C:G substrate, respectively. Klimasauskas and Roberts^43^ compiled data for M. **Hha*I* binding to a series of mismatches, and they compared aggregated data in the form of a relative affinity parameter R for the various mismatches and normalized the data to a reference data set. A correlation with our *K*_eq_ data is given in Figure 6C. It should be noted, as they state in their paper, that the M. **Hha*I* data are compiled under numerous sample conditions including variations in temperature, buffer, and concentrations. Likewise, our values are taken as averages at 25 °C of the data from our multiple sequences. Even with these caveats, a strong correlation is present. The transcription factor CCAAT/enhancer binding protein (CEBPβ) has demonstrated enhanced binding to mismatched substrates.^30,31^ A strong quantitative correlation between *K_D_* and the backbone *K*_eq_ is shown in Figure 6D.

All data for TDG and MBD4 were obtained by the same research group,^22−25^ and so should have a high degree of internal consistency, using saturating enzyme concentrations to measure single turnover kinetics in triplicate. The reactions were analyzed by HPLC assays and nonlinear least squares fits of the integrated intensities. CEBPβ *K*_D_ values were obtained by fluorescence assays in quadruplicate where 6-carboxyfluorescein-labeled DNA was incubated in increasing amounts of protein.^30^ The M. **Hha*I* binding experiments were evaluated with gel mobility shift assays. The authors of that work^43^ admit that there was variation in the absolute values observed in their experiments, but their relative numbers were very reproducible. So while the kinetic analysis will vary for the different enzymes, within the studies of particular enzymes, there is excellent consistency in data.

The quantitative relationship between the thermodynamic and kinetic parameters should also be considered in the context of important enzyme–DNA interactions. Protein–phosphate contacts in nucleotide flipping enzymes is a common motif.^12^ In particular, a recent TDG–substrate complex, R110, demonstrates a contact with the phosphate 5′ to the mismatched T, and in a TDG–product complex, R275 has direct contacts with the two phosphates flanking the target where the side chain fills the space vacated by the flipped base and may serve the role of stabilizing the flipped positioning.^44^ The latter contact is likely related to the phosphate “pinch” that occurs as a part of nucleotide flipping.^45^ Mutational analysis shows that *k*_max_ is significantly reduced in R110A^44^ and R275A^46^ mutants, indicating the importance of these contacts. From these results, it was concluded that the R275 promotes and/or stabilizes nucleotide flipping by TDG.^46^ Similarly, there is an important arginine (R165) residue on M. **Hha*I* that makes phosphate contacts during complexation with its substrate,^47^ and there are several phosphate interactions when complexed with mismatched DNA.^13^ It is conceivable that the energy differences in the backbone facilitate the role of the arginine in recognition and/or nucleotide flipping.

The proteins discussed herein are not the only ones that bind T:G mismatches, nor are TDG and MBD4 the only enzymes that repair T:G mismatches, so it is interesting to compare and contrast our results more broadly. For example, the enzyme MutS and its homologues operate in dimers to repair single base mismatches (not just T:G) and 1–2 base insertion-deletion loops during the process of Mismatch Repair (MMR).^48,49^ MutS and DNA glycosylases have different chemical operations, with glycosylases binding and removing the aberrant base by cleaving the glycosidic bond^2,49^ and MutS recognizing the mismatch, acting as a molecular clamp,^48,49^ switch,^50^ or to coordinate other enzymes.^51^ MutS enzymes repair a variety of mismatches,^48^ whereas TDG and MBD4 are active on a small set of substrates. The crystal structure of *E. coli* MutS bound to a DNA containing a T:G mismatch offers several points of comparison.^52^ While MutS imposes a significant bend or kink to the overall DNA helix, there is no nucleotide flipping involving the target base pair as there is for TDG and MBD4. Finally, BER enzymes are generally not ATP-dependent, whereas MutS explicitly binds ATP.

The presence and use of ATP is an interesting one. As glycosylases do not generally require ATP, it is not unreasonable to speculate that they utilize the perturbed energetic landscape of the DNA backbone to promote and utilize nucleotide flipping. Only one known nucleotide flipping enzyme uses ATP,^53^ a methyltransferase from a fungus, and the first known from the fungal and protist kingdoms. Other DNA-binding enzymes such as MutS might use ATP for the energy necessary to promote the structural rearrangements necessary for catalysis, and therefore backbone perturbations in free substrates of ATP-using enzymes may not be observed.

MutS does have sequence dependence in its affinities for its T:G mismatch substrates,^54^ where several sequences with different flanking nucleotides were analyzed for dissociation constants *K*_D_ and *k*_off_. This work did not evaluate a set of sequences where the only changes to the sequences are the four possible base pairs flanking the mismatch on the same side (5′ to the G), making it impossible to generate a quantitative correlation. In contrast to TDG, MutS appears to have a directional dependence as it is tracking the DNA during repair, and therefore more sensitive to the base pair on the opposite side of the mismatch. TDG has only modest dependence of the base to the 5′side of the T (Alex Drohat, personal communication). There are some very qualitative trends for MutS, where generally the sequences with a pyrimidine as the 5′ neighbor to the base-paired G (CpG:T and TpG:T) have the largest *K*_D_ and *k*_off_ values and the other sequences (GpG:T and ApG:T) have the smallest values. The trends observed for MutS, where the CpG:T and TpG:T sequences are the weakest substrates, are the opposite trends we observe for the other enzymes discussed.

The very short patch (VSP) repair endonuclease also repairs T:G mismatches. From the crystal structure of a VSP-DNA complex, it is also clear that nucleotide flipping is not utilized for recognition.^55^ Additionally, VSP has a canonical recognition sequence of CC(A/T)GG where the internal C is repaired if converted to 5meC and subsequently T,^49,56^ rather than recognizing T:G mismatches in all sequence contexts as TDG and MBD4 do. Rather than cleaving the glycosidic bond, VSP endonuclease creates a nick to the 5′ side of the mismatches T and also utilizes two Mg^2+^ ions during its repair, whereas TDG and MBD4 do not require divalent metals. As neither MutS nor VSP uses nucleotide flipping and different processes in their repair, this adds additional credence to the relevance of the backbone conformation to the nucleotide flipping reaction coordinate during repair.

When considering interactions and molecular properties during biomolecular recognition, it is important to consider static or average structural characteristics, as well as dynamic or thermodynamic properties, and the interplay between them. An important initial observation is that the sequences with the highest activity as substrates have pyrimidines as the neighboring nucleotide 5′ to the paired G (i.e., CpG:T and TpG:T). It is not unreasonable to think that the nature of the base itself, hydrogen bonding, sterics, or stacking energies plays a role in recognition or repair. Indeed, T:G mismatches have uniformly been shown to have wobble base pairs both from solution NMR^57−61^ and X-ray crystallography.^62^ While offering interesting insights, only three of the NMR structures provided analysis of helical parameters.^59−61^ Two of the NMR structures^59,60^ were also evaluated by Afek et al.,^11^ but an additional structure^61^ is also available. It seems that the presence of a wobble base pair alone is insufficient to account for the variation in the protein activity discussed herein. Unfortunately, while the three sequences analyzed by NMR include CpG:T, TpG:T, and ApG:T sequence contexts, there seems to be no representation of the GpG:T in the literature, only one sequence has analyzed the canonical control sequence by NMR, and all three overall sequences are different. This limits the utility of any detailed analysis of the helical properties by programs such as CURVES+.^63^ Nevertheless, we found no obvious trends in any helical parameters while admitting further analysis would be beneficial.

Likewise, we can attempt to visualize the base stacking from the three NMR structures. In Figure S20, the dinucleotides from these three structures containing the mismatches are displayed, and displaced stacking of the T is observed in all, presumably due to the wobble base pair. However, there appears to be no overall difference in the qualitative amount of unstacking that would seem to correlate with the dramatic difference in enzyme activity for the different neighboring nucleotides.

An extensive study^64^ using NMR chemical shift perturbation (CSP) and relaxation dispersion (RD), X-ray structure surveying, and MD found low populations of Watson–Crick-like base pairs in T:G mismatches DNA with two different sequence contexts (CpG:T and GpG:T). Additionally, they observed significant ^13^C chemical shift perturbations in the C3′ and C4′ carbons in the mismatched base pair as well as the neighboring nucleotides on the 3′ side of the T and 5′ side of the base-paired G, consistent with our results for the DNA phosphates (which they state require ^31^P studies to characterize). Their work combined with our current and previous results^14,15^ present a strong case for a significant perturbation of the sugar-phosphate backbone properties in the vicinity of a T:G mismatch. They also conclude from crystal structure surveys and MD that there is a shift to lower BII near the mismatch. This is only in partial agreement with our results where we instead observe the stepwise pattern of low BII to high BII flanking the mismatched T, as shown in Figure 3. Additionally, their results do not follow the same sequence-dependent pattern as ours, where the largest CSP is in the GpG:T context rather than the CpG:T, indicating further analysis will be beneficial.

Base stacking energy and melting thermodynamics could also play a role in recognition and/or nucleotide flipping in DNA repair and be related to the backbone conformations. Allawi and Santalucia^65^ determined from melting studies of T:G mismatched DNA that the ApG:T sequence context has the largest positive Δ*G*_37_^◦^, while the CpG:T has the largest negative Δ*G*_37_^◦^. This result qualitatively matches our trend, though we differ in relative values for the TpG:T and GpG:T contexts. Likewise, additional analysis showed that the largest stacking energy for T:G mismatches is for the ApG:T context and the smallest is for the CpG:T context.^66^ It is satisfying to see connections between these varied properties, but further work is necessary to quantify their connections to each other and which play the primary role(s) in DNA repair and damage recognition. We feel our work demonstrates a compelling, quantitative relationship between the backbone thermodynamics and enzyme activity and binding.

The altered backbone dynamic properties in mismatches will perhaps be manifested in other experimental properties such as base-pair opening parameters, given that open and closed states would likely have different backbone conformations, and the rate constants for opening and closing might potentially correlate with backbone conformational populations. From MD studies of U:A base pairs, hydrogen bond disruption is accompanied by conformational changes in the backbone including inversion of BI and BII states.^67^ Imino exchange studies in DNA containing a T:G mismatch conclude that the equilibrium constant for base pair opening is increased up to 4000-fold relative to a C:G and the base pair lifetimes are reduced by 2 orders of magnitude. Significant changes to base-pair opening enthalpy and enthalpies of activation for base-pair opening were also obtained.^68^ Other imino studies with DNA in the presence of uracil DNA glycosylase (UNG), a DNA glycosylase which also repairs T:G mismatches utilizing nucleotide flipping, showed that UNG increases the equilibrium constant for opening of T:A base pair by 2 orders of magnitude.^69^ Imino exchange can occur with only a 30-degree opening relative to the base-paired state,^70,71^ so one must be cautious extrapolating these results to be relevant to a fully flipped reaction coordinate. ^19^F NMR studies concluded that U:G base pairs were predominantly stacked in the duplex, and the nucleotide flipping by TDG occurred on a relatively slow timescale (<9000 s^−1^).^24^ All of these results suggest further work will be necessary to connect a relationship between the backbone and base-pair opening properties.

What role, then, does the perturbed backbone equilibrium play in the repair and/or nucleotide flipping process? DNA glycosylases do not require ATP for their function (unlike other repair enzymes such as MutS) but rather use diffusion as they search for their target. These same stepwise high-BI-to-high-BII phosphate conformations flanking the target appear to be an energetic or conformational cue to the enzymes to stop, bind, interrogate, and initiate catalysis. Additionally, given the consistently high correlation with activity in nucleotide flipping enzymes, the perturbed energetic landscape of the backbone interconversion likely provides an energetic preference for the flipping reaction coordinate. Regardless of the specific role or roles, there is nevertheless a strong correlation with enzyme activity, and their direct involvement in protein–DNA interaction and recognition of the preferred substrate is strongly supported by our results. Further investigations will evaluate additional support for the energetic component biomolecular recognition.

## Supporting Information Available

Complete chemical shift assignments, representative NMR spectra, and additional tables of %BII (PDF)

## References

[ref1] Wallace S. S. Base excision repair: A critical player in many games. DNA Repair 2014, 19, 14–26. 10.1016/j.dnarep.2014.03.030. 24780558 PMC4100245

[ref2] Jacobs A. L.; Schär P. DNA glycosylases: in DNA repair and beyond. Chromosoma 2012, 121, 1–20. 10.1007/s00412-011-0347-4. 22048164 PMC3260424

[ref3] Hitomi K.; Iwai S.; Tainer J. A. The intricate structural chemistry of base excision repair machinery: Implications for DNA damage recognition, removal, and repair. DNA Repair 2007, 6, 410–418. 10.1016/j.dnarep.2006.10.004. 17208522

[ref4] Grundy G. J.; Parsons J. L. Base excision repair and its implications to cancer therapy. Essays in Biochemistry 2020, 64, 831–843. 10.1042/EBC20200013. 32648895 PMC7588666

[ref5] Yang W. Structure and mechanism for DNA lesion recognition. Cell Res. 2008, 18, 184–197. 10.1038/cr.2007.116. 18157156

[ref6] Klimasauskas S.; Kumar S.; Roberts R. J.; Cheng X. *HhaI* Methyltransferase Flips Its Target Base Out of the DNA Helix. Cell 1994, 76, 357–369. 10.1016/0092-8674(94)90342-5. 8293469

[ref7] Hong S.; Cheng X. DNA Base Flipping: A General Mechanism for Writing, Reading, and Erasing DNA Modifications. Adv. Exp. Med. Biol. 2016, 945, 321–341. 27826845 10.1007/978-3-319-43624-1_14 PMC5542066

[ref8] Esadze A.; Stivers J. T. Facilitated Diffusion Mechanisms in DNA Base Excision Repair and Transcriptional Activation. Chem. Rev. 2018, 118, 11298–11323. 10.1021/acs.chemrev.8b00513. 30379068 PMC6504930

[ref9] Hedglin M.; Zhang Y.; O’Brien P. J. Probing the DNA Structural Requirements for Facilitated Diffusion. Biochemistry 2015, 54, 557–566. 10.1021/bi5013707. 25495964 PMC4303293

[ref10] Schonhoft J. D.; Stivers J. T. Timing facilitated site transfer of an enzyme on DNA. Nature Chemical Biology 2012, 8, 205–210. 10.1038/nchembio.764. 22231272 PMC3262087

[ref11] Afek A.; Shi H.; Rangadurai A.; Sahay H.; Senitzki A.; Xhani S.; Fang M.; Salinas R.; Mielko Z.; Pufall M. A.; Poon G. M. K.; Haran T. E.; Schumacher M. A.; Al-Hashimi H. M.; Gordan R. DNA mismatches reveal conformational penalties in protein-DNA recognition. Nature 2020, 587, 291–296. 10.1038/s41586-020-2843-2. 33087930 PMC7666076

[ref12] Estabrook R. A.; Lipson R.; Hopkins B.; Reich N. The Coupling of Tight DNA Binding and Base Flipping. J. Bio. Chem. 2004, 279, 31419–31428. 10.1074/jbc.M402950200. 15143064

[ref13] O’Gara M.; Horton J. R.; Roberts R. J.; Cheng X. Structures of *HhaI* methyltranferase complexed with substrates containing mismatches at the target base. Nature 1998, 5, 872–877. 10.1038/2312 9783745

[ref14] Westwood M. N.; Ljunggren K. D.; Boyd B.; Becker J.; Dwyer T.; Meints G. A. Single Base Lesions and Mismatches Alter the Backbone Conformational Dynamics in DNA. Biochemistry 2021, 60, 873–885. 10.1021/acs.biochem.0c00784. 33689312

[ref15] Westwood M. N.; Johnson C. C.; Oyler N. A.; Meints G. A. Kinetics and thermodynamics of BI-BII interconversion altered by T:G mismatches. Biophysical J. 2022, 121, 1691–1703. 10.1016/j.bpj.2022.03.031. PMC9117933 35367235

[ref16] Bellacosa A.; Drohat A. C. Role of base excision repair in maintaining the genetic and epigenetic integrity of CpG sites. DNA Repair 2015, 32, 33–42. 10.1016/j.dnarep.2015.04.011. 26021671 PMC4903958

[ref17] Cortázar D.; Kunz C.; Saito Y.; Steinacher R.; Schär P. The enigmatic thymine DNA glycosylase. DNA Repair 2007, 6, 489–504. 10.1016/j.dnarep.2006.10.013. 17116428

[ref18] Hendrich B.; Hardeland U.; Ng H.-H.; Jiricny J.; Bird A. The thymine glycosylase MBD4 can bind to the product of deamination at methylated CpG sites. Nature 1999, 401, 301–304. 10.1038/45843. 10499592

[ref19] Abu M.; Waters T. R. The Main Role of Human Thymine-DNA Glycosylase Is Removal of Thymine Produced by Deamination of 5-Methylcytosine and NOT Removal of Ethenocytosine. J. Biol. Chem 2003, 278, 8739–8744. 10.1074/jbc.M211084200. 12493755

[ref20] Waters T. R.; Swann P. F. Kinetics of the Action of Thymine DNA Glycosylase. J. Biol. Chem 1998, 273, 20007–20014. 10.1074/jbc.273.32.20007. 9685338

[ref21] Sibghat-Ullah; Gallinari P.; Xu Y.-Z.; Goodman M. F.; Bloom L. B.; Jiricny J.; Day R. S. III Base Analog and Neighboring Base Effects on Substrate Specificity of Recombinant Human G:T Mismatch-Specific Thymine DNA-Glycosylase. Biochemistry 1996, 35, 12926–12932. 10.1021/bi961022u. 8841138

[ref22] Bennett M. T.; Rodgers M. T.; Hebert A. S.; Ruslander L. E.; Eisele L.; Drohat A. C. Specificity of Human Thymine DNA Glycosylase Depends on N-Gycosidic Bond Stability. J. Am. Chem. Soc. 2006, 128, 12510–12519. 10.1021/ja0634829. 16984202 PMC2809119

[ref23] Morgan M. T.; Bennett M. T.; Drohat A. C. Excision of 5-Halogenated Uracils by Human Thymine DNA Glycosylase. Robust activity for DNA contexts other than CpG. J. Bio. Chem. 2007, 282, 27578–27586. 10.1074/jbc.M704253200. 17602166 PMC2818988

[ref24] Dow B. J.; Malik S. S.; Drohat A. C. Defining the Role of Nucleotide Flipping in Enzyme Specificity Using ^19^F NMR. J. Am. Chem. Soc. 2019, 141, 4952–4962. 10.1021/jacs.9b00146. 30841696 PMC6437012

[ref25] Manvilla B. A.; Maiti A.; Begley M. C.; Toth E. A.; Drohat A. C. Crystal Structure of Human Methyl-Binding Domani IV Glycosylase Bound to Abasic DNA. J. Mol. Biol. 2012, 420, 164–175. 10.1016/j.jmb.2012.04.028. 22560993 PMC3372577

[ref26] Sanchez A. M.; Volk D. E.; Gorenstein D. G.; Lloyd R. S. Initiation of repair of A/G mismatches is modulated by sequence context. DNA Repair 2003, 2, 863–878. 10.1016/S1568-7864(03)00067-3. 12893083

[ref27] Wolfe A. E.; O’Brien P. J. Kinetic Mechanism for the Flipping and Excision of 1,N6-Ethenoadnine by Human Alkyladenine DNA Glycosylase. Biochemistry 2009, 48, 11357–11369. 10.1021/bi9015082. 19883114 PMC2792197

[ref28] Youngblood B.; Buller F.; Riech N. O. Determinants of Sequence-Specific DNA Methylation: Target Recognition and Catalysis Are Coupled in M. *Hha*I. Biochemistry 2006, 45, 15563–15573. 10.1021/bi061414t. 17176077

[ref29] Lin C.-C.; Chen Y.-P.; Yang W. -Z.; Shen J. C. K.; Yuan H. S. Structural insights into CpG-specific DNA methylation by human DNA methyltransferase 3B. Nucleic Acids Res. 2020, 48, 3949–3961. 10.1093/nar/gkaa111. 32083663 PMC7144912

[ref30] Yang J.; Horton J. R.; Akdemir K. C.; Li J.; Huang Y.; Kumar J.; Blumenthal R. B.; Zhang X.; Cheng X. Preferential CEBP binding to T:G mismatches and increased C-to-T human somatic mutations. Nucleic Acids Res. 2021, 49, 5084–5094. 10.1093/nar/gkab276. 33877329 PMC8136768

[ref31] Ershova A. S.; Eliseeva I. A.; Kikonov O. S.; Fedorova A. D.; Vorontsov I. E.; Papatsenko D.; Kulakovsky I. V. Enhanced C/EBP binding to G:T mismatches facilitates fixation of CpG mutations in cancer and adult stem cells. Cell Rep. 2021, 35, 109221 10.1016/j.celrep.2021.109221. 34107262

[ref32] Gorenstein D. G. ^31^P NMR of DNA. Meth. Enzym. 1992, 211, 254–286. 1406310 10.1016/0076-6879(92)11016-c

[ref33] Gorenstein D. G. Conformation and Dynamics of DNA and Protein-DNA Complexes by ^31^P NMR. Chem. Rev. 1994, 94, 1315–1338. 10.1021/cr00029a007.

[ref34] Heddi B.; Foloppe N.; Bouchemal N.; Hantz E.; Hartmann B. Quantification of DNA BI/BII Backbone States in Solution. Implications for DNA Overall Structure and Recognition. J. Am. Chem. Soc. 2006, 128, 9170–9177. 10.1021/ja061686j. 16834390

[ref35] Tian Y.; Kayatta M.; Shultis K.; Gonzalez A.; Mueller L. J.; Hatcher M. E. ^31^P NMR Investigation of Backbone Dynamics in DNA Binding Sites. J. Phys. Chem. B 2009, 113, 2596–2603. 10.1021/jp711203m. 18717548 PMC2711773

[ref36] Hare D. R.; Wemmer D. E.; Chou S.-H.; Drobny G.; Reid B. R. Assignment of the Non-Exchangeable Proton Resonances of d(C-G-C-G-A-A-T-T-C-G-C-G) Using Two-dimensional Nuclear Magnetic Resonance Methods. J. Mol. Biol. 1983, 171, 319–336. 10.1016/0022-2836(83)90096-7. 6317867

[ref37] Wüthrich K. NMR of Proteins and Nucleic Acids, John Wiley & Sons: New York, 1986.

[ref38] Evans J. N. S. Biomolecular NMR Spectroscopy; Oxford University Press, Inc.: New York, 1995.

[ref39] Gottlieb H. E.; Kotlar V.; Nudelman A. NMR Chemical Shifts of Common Laboratory Solvents as Trace Impurities. J. Org. Chem. 1997, 62, 7512–7515. 10.1021/jo971176v. 11671879

[ref40] Mazurek A.; Johnson C. N.; Germann M. W.; Fishel R. Sequence context effect for hMSH2-hMSH6 mismatch-dependent activation. Proc. Natl. Acad. Sci. U.S.A. 2009, 106, 4177–4182. 10.1073/pnas.0808572106. 19237577 PMC2657375

[ref41] Hendershot J. M.; O’Brien P. J. Transient Kinetic Methods for Mechanistic Characterization of DNA Binding and Nucleotide Flipping. Methods Enz. 2017, 592, 377–415. 10.1016/bs.mie.2017.04.003. 28668128

[ref42] Yang A. S.; Shen J.-C.; Zingg J.-M.; Mi S.; Jones P. A. *HhaI* and *HpaII* DNA methyltransferases bind DNA mismatches, methylate uracil and block DNA repair. Nucleic Acids Res. 1995, 23, 1380–1387. 10.1093/nar/23.8.1380. 7753629 PMC306865

[ref43] Klimasauskas S.; Roberts R. J. M. *HhaI* binds tightly to substrates containing mismatches at the target base. Nucleic Acids Res. 1995, 23, 1388–1395. 10.1093/nar/23.8.1388. 7753630 PMC306866

[ref44] Coey C. T.; Malik S. S.; Pidugu L. S.; Varney K. M.; Pozharski E.; Drohat A. C. Structural basis of damage recognition by thymine DNA glycosylase: Key roles for N-terminal residues. Nucleic Acids Res. 2016, 44, 10248–10258. 10.1093/nar/gkw768. 27580719 PMC5137436

[ref45] Maiti A.; Morgan M. T.; Pozharski E.; Drohat A. C. Crystal structure of human thymine DNA glycosylase bound to DNA elucidates sequence-specific mismatch recognition. Proc. Natl. Acad. Sci. U.S.A. 2008, 105, 8890–8895. 10.1073/pnas.0711061105. 18587051 PMC2449336

[ref46] Maiti A.; Morgan M. T.; Drohat A. C. Role of Two Strictly Conserved Residues in Nucleotide Flipping and N-Glycosidic Bond Cleavage by Human Thymine DNA Glycosylase. J. Biol. Chem. 2009, 284, 36680–36688. 10.1074/jbc.M109.062356. 19880517 PMC2794782

[ref47] Shieh F.-K.; Youngblood B.; Reich N. O. The Role of Arg165 Towards Base Flipping, Base Stabilization and Catalysis in M. *Hha*I. J. Mol. Biol. 2006, 362, 516–527. 10.1016/j.jmb.2006.07.030. 16926025

[ref48] Kunkel T. A.; Erie D. A. DNA Mismatch Repair. Annu. Rev. Biochem. 2005, 74, 681–710. 10.1146/annurev.biochem.74.082803.133243. 15952900

[ref49] Friedberg E. C.; Walker G. C.; Siede W.; Wood R. D.; Schultz R. A.; Ellenberger T. DNA Repair and Mutagenesis, 2nd ed.; ASM Press: Washington, D.C., 2006.

[ref50] Lebbink J. H. G.; Fish A.; Reumer A.; Natrajan G.; Winterwerp H. H.; Sixma T. K. Magnesium Coordination Controls the Molecular Switch Function of DNA Mismatch repair Protein MutS. J. Biol. Chem. 2010, 285, 13131–13141. 10.1074/jbc.M109.066001. 20167596 PMC2857095

[ref51] Leblanc S. J.; Gauer J. W.; Hao P.; Case B. C.; Hingorani M. M.; Wininger K. R.; Erie D. A. Coordianted protein and DNA conformational changes govern mismatch repair initiation y MutS. Nucleic Acids Res. 2018, 46, 10782–10795. 10.1093/nar/gky865. 30272207 PMC6237781

[ref52] Lamers M. H.; Perrakis A.; Enzlin J. H.; Winterwep H. H. K.; de Wind N.; Sixma T. K. The crystal structure of DNA mismatch repair protein MutS binding to a G·T mismatch. Nature 2000, 407, 711–717. 10.1038/35037523. 11048711

[ref53] Wang J.; Catania S.; Wang C.; de la Cruz M. J.; Rao B.; Madhani H. D.; Patel D. J. Structural insights into DNMT5-mediated ATP-dependent high-fidelity epigenome maintenance. Mol. Cell. 2022, 82, 1186–1198. 10.1016/j.molcel.2022.01.028. 35202575 PMC8956514

[ref54] Groothuizen F. S.; Fish A.; Petoukhov M. V.; Reumer A.; Manelyte L.; Winterwerp H. H. K.; Marinus M. G.; Lebbink J. H. G.; Svergun D. I.; Friedhoff P.; Sixma T. K. Using stable MutS dimers and tetramers to quantitatively analyze DNA mismatch recognition and sliding clamp formation. Nucleic Acids Res. 2013, 41, 8166–8181. 10.1093/nar/gkt582. 23821665 PMC3783165

[ref55] Tsutokawa S. E.; Jingami H.; Morikawa K. Recognition of a TG Mismatch: The Crystal Structure of Very Short Patch repair Endonuclease in Complex with a DNA Duplex. Cell 1999, 99, 615–623. 10.1016/S0092-8674(00)81550-0. 10612397

[ref56] Tsutakawa S. E.; Muto T.; Kawate T.; Jingami H.; Kunishima N.; Ariyoshi M.; Kohda D.; Nakagawa M.; Morikawa K. Crystallographic and Functional Studies of Very Short Patch Repair Endonuclease. Mol. Cell 1999, 3, 621–628. 10.1016/S1097-2765(00)80355-X. 10360178

[ref57] Patel D. J.; Kozlowski S. A.; Marky L. A.; Rice J. A.; Broka C.; Dallas C.; Itakura K.; Breslauer K. J. Structure, Dynamics, and Energetics of Deoxyguanosine-Thymidine Wobble Base Pair Formation in the Self-Complementary d(CGTGAATTCGCG) Duplex in Solution. Biochemistry 1982, 21, 437–444. 10.1021/bi00532a003. 7066295

[ref58] Hare D.; Shapiro L.; Patel D. J. Wobble dG•dT Pairing in Right-Handed DNA: Solution Conformation of the d(C-G-T-G-A-A-T-T-C-G-C-G) Duplex Deduced from Distance Geometry Analysis of Nuclear Overhauser Effect Spectra. Biochemistry 1986, 25, 7445–7456. 10.1021/bi00371a029. 3801424

[ref59] Allawi H. T.; SantaLucia J. Jr. NMR solution structure of a DNA dodecamer containing single G•T mismatches. Nucleic. Acids. Res. 1998, 26, 4925–4934. 10.1093/nar/26.21.4925. 9776755 PMC147937

[ref60] Isaacs R. J.; Rayens W. S.; Spielmann H. P. Structural Differences in the NOE-derived Structure of G-T Mismatched DNA Relative to Normal DNA are Correlated with Differences in ^13^C Relaxation-based Internal Dynamics. J. Mol. Biol. 2002, 319, 191–207. 10.1016/S0022-2836(02)00265-6. 12051946

[ref61] Pfaff D. A.; Clarke K. M.; Parr T. A.; Cole J. M.; Beierstanger B. H.; Tahmassebi D. C.; Dwyer T. J. Solution Structure of a DNA Duplex Containing a Guanine-Difluorotoluene Pair: A Wobble Pair without Hydrogen Bonding?. J. Am. Chem. Soc. 2008, 130, 4869–4878. 10.1021/ja7103608. 18341343

[ref62] Hunter W. N.; Brown T.; Kneale G.; Anand N. N.; Rabinovich D.; Kennard O. The Structure of Guanosine-Thymidine Mismatches in B-DNA at 2.5 Å Resolution. J. Biol. Chem. 1987, 262, 9962–9970. 10.1016/S0021-9258(18)61060-9. 3611072

[ref63] Lavery R.; Moakher M.; Maddocks J. H.; Petkeviciute D.; Zakrzewska K. CURVES+ web server for analyzing and visualizing the helical, backbone and groove parameters of nucleic acid structures. Nucleic Acids Res. 2009, 37, 5917–5929. 10.1093/nar/gkp608. 21558323 PMC3125750

[ref64] Rangadurai A.; Szymanski E. S.; Kimsey I.; Shi H.; al-Hashimi H. M. Probing conformational transitions towards mutagenic Watson-Crick-like G·T mismatches using off-resonance sugar carbon R1ρ relaxation dispersion. J. Biomol. NMR 2020, 74, 457–471. 10.1007/s10858-020-00337-7. 32789613 PMC7508749

[ref65] Allawi H. T.; SantaLucia J. Jr. Thermodynamics and NMR of Internal G·T Mismatches in DNA. Biochemistry 1997, 36, 10581–10594. 10.1021/bi962590c. 9265640

[ref66] Oliveira L. M.; Long A. S.; Brown T.; Fox K. R.; Weber G. Melting temperature measurement and mesoscopic evaluation of single, double, and triple DNA mismatches. Chem. Sci. 2020, 11, 8273–8287. 10.1039/D0SC01700K. 34094181 PMC8163305

[ref67] Fadda E.; Pomès R. On the molecular basis of uracil recognition in DNA: comparative study of T-A versus U-A structure, dynamics and open base pair kinetics. Nucleic Acids Res. 2011, 39, 767–780. 10.1093/nar/gkq812. 20876689 PMC3025553

[ref68] Moe J. G.; Russu I. M. Proton exchange and base-pair opening kinetics in 5′-d(CGCGAATTCGCG)-3′ and related dodecamers. Nucleic Acids Res. 1990, 18, 821–827. 10.1093/nar/18.4.821. 2156233 PMC330333

[ref69] Cao C.; Jiang Y. L.; Stivers J. T.; Song F. Dynamic opening of DNA during the enzymatic search for a damaged base. Nat. Struct. Mol. Biol. 2004, 11, 1230–1236. 10.1038/nsmb864. 15558051

[ref70] Priyakumar U. D.; MacKerell A. D. Jr. Computational Approaches for Investigating Base Flipping in Oligonucleotides. Chem. Rev. 2006, 106, 489–505. 10.1021/cr040475z. 16464016

[ref71] Yin Y.; Yang L.; Zheng G.; Gu C.; Yi C.; He C.; Gao Y. Q.; Zhao X. S. Dynamics of spontaneous flipping of a mismatched base in DNA duplex. Proc. Natl. Acad. Sci. U.S.A. 2014, 111, 8043–8048. 10.1073/pnas.1400667111. 24843124 PMC4050552

