# Supplementary material for: Backbone Conformational Equilibrium in Mismatched DNA Correlates with Enzyme Activity
Source: Biochemistry. 2023 Sep 12;62(19):2816–27. doi: 10.1021/acs.biochem.3c00230 (PMC10552547; doi:10.1021/acs.biochem.3c00230)
Supplement: Supplementary file 1 — bi3c00230_si_001.pdf [file bi3c00230_si_001.pdf]

DNA Backbone Conformational Equilibrium Correlates with Enzyme Activity

M. N. Westwood, † A. Pilarski,* C. Johnson,* S. Mamoud* and G. A. Meints*

*Department of Chemistry and Biochemistry, Missouri State University, 901 S. National Ave., Springfield, MO, USA 65897

† Biophysics Program, University of Michigan, 930 N. University Avenue, Ann Arbor, MI 48109, USA

*E-mail address of the corresponding author

garymeints@missouristate.edu

Tel 417 836 6291

Fax 417 836 5507

Supplemental Data

8mer Control DNA

8mer Control ^{1}H Assignments

**8mer Control**	H8/H6	H2/H5/Me	1'	2'	2''	3'	4′
G1	7.90		5.59	2.55	2.74	4.85	4.21
A2	8.20		6.06	2.81	2.92	5.07	4.43
G3	7.69		5.82	2.55	2.68	4.99	4.40
C4	7.26	5.22	5.7	2.04	2.41	4.84	4.19
G5	7.92		6.03	2.72	2.82	4.98	4.4
T6	7.31	1.46	6.07	2.15	2.61	4.86	4.24
T7	7.43	1.70	6.18	2.19	2.58	4.89	4.14
C8	7.54	5.58	6.30	2.31	2.31	4.60	4.02
G9	7.89		5.55	2.57	2.78	4.84	4.21
A10	8.26		5.93	2.82	2.93	5.08	4.43
A11	8.16		6.17	2.63	2.88	5.04	4.48
C12	7.16	5.15	5.59	1.89	2.32	4.80	4.14
G13	7.85		5.89	2.68	2.7	4.96	4.37
C14	7.42	5.33	5.94	2.11	2.53	4.72	4.22
T15	7.50	1.68	6.13	2.2	2.55	4.88	4.16
C16	7.57	5.61	6.30	2.31	2.31	4.60	4.02

Table S1. ^{1}H Assignments for 8mer DNA at 283K. The δH values have been referenced to the temperature-dependent HDO signal per Gottlieb et al.

<u>8mer Control ^{31}P Assignments</u>

DNA position	278K	283K	288K	293K
G1pA2	-0.42	-0.41	-0.41	-0.39
A2pG3	-0.50	-0.51	-0.51	-0.50
G3pC4	-0.42	-0.41	-0.41	-0.41
C4pG5	-0.34	-0.35	-0.36	-0.37
G5pT6	-0.66	-0.66	-0.66	-0.65
T6pT7	-0.61	-0.62	-0.63	-0.62
T7pC8	-0.29	-0.29	-0.26	-0.30
G9pA10	-0.42	-0.41	-0.41	-0.39
A10pA11	-0.50	-0.51	-0.51	-0.50
A11pC12	-0.52	-0.53	-0.53	-0.52
C12pG13	-0.42	-0.41	-0.41	-0.41
G13pC14	-0.30	-0.31	-0.34	-0.34
C14pT15	-0.69	-0.69	-0.69	-0.68
T15pC16	-0.29	-0.25	-0.26	-0.30

Table S2. ^{31}P assignments for 8mer control DNA as a function of temperature. The δP values have been externally referenced to H_3PO_4 at 0.00 ppm via coaxial insert.

__8mer Control NOESY Spectra__

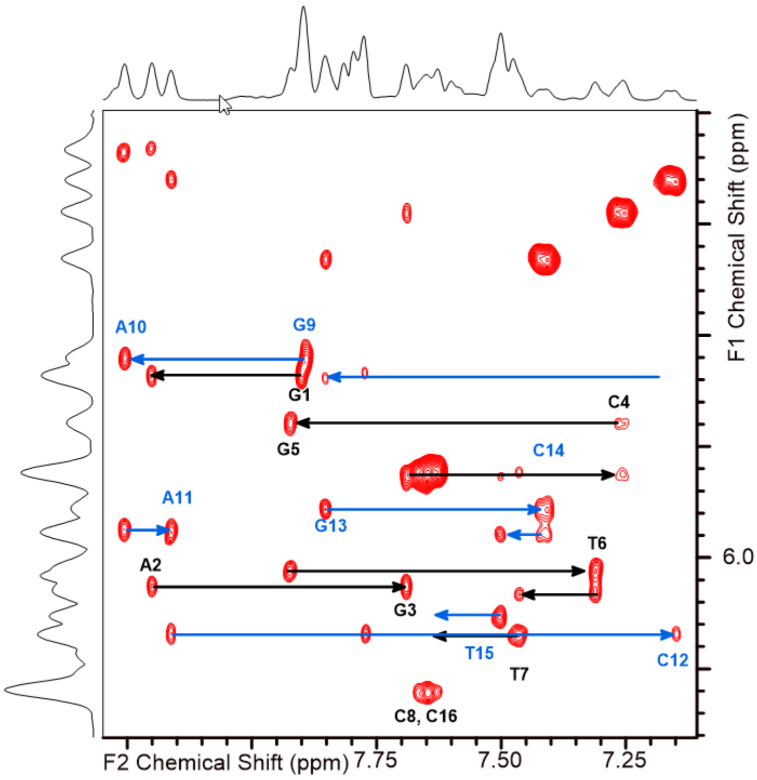

Figure S1. Fingerprint region of the NOESY spectrum of 8mer DNA showing the aromatic–H1′ intranucleotide and sequential connectivities at 283K. All labels are shown as the respective intranucleotide aromatic-1' crosspeak for the indicated nucleotide. The black lines represent the strand containing the mismatched T. The blue lines represent the strand containing the base-paired G. The δH values have been referenced to the temperature-dependent HDO signal per Gottlieb et al.

8mer Control HSQC Spectra

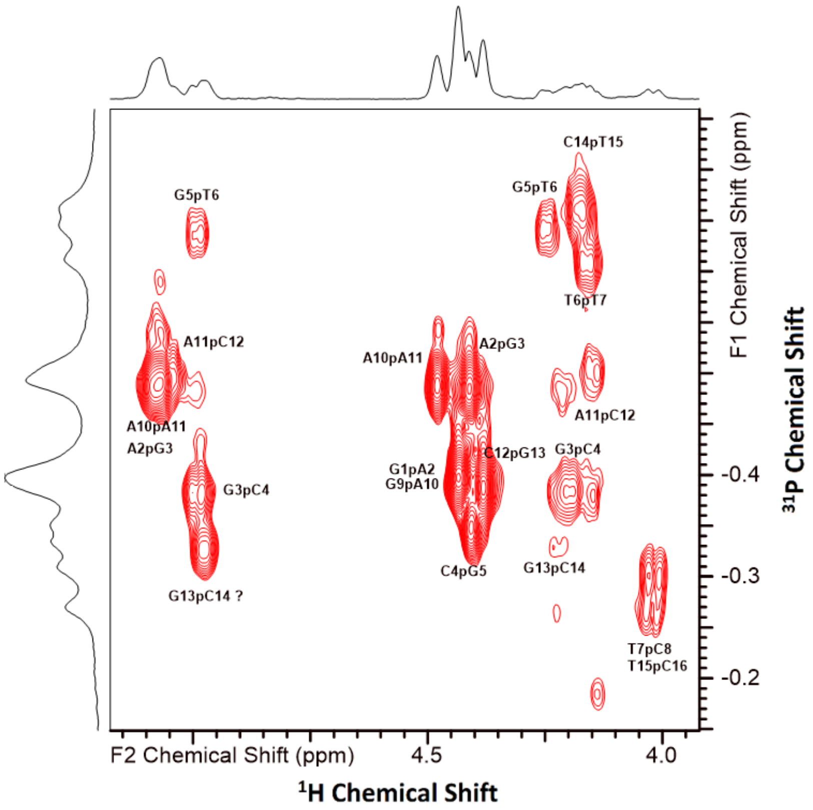

Figure S2. HSQC spectrum of the 8mer control sequence at 293K indicating the H4' to P crosspeaks with the internucleotide phosphate indicated. Note the F2 axis (^{31}P) has been externally referenced to H_3PO_4 at 0.00 ppm via coaxial insert. The F1 axis represents the 4' proton for the 5' nucleotide of the dinucleotide pair.

8mer Control 1D ^{31}P Temperature Study

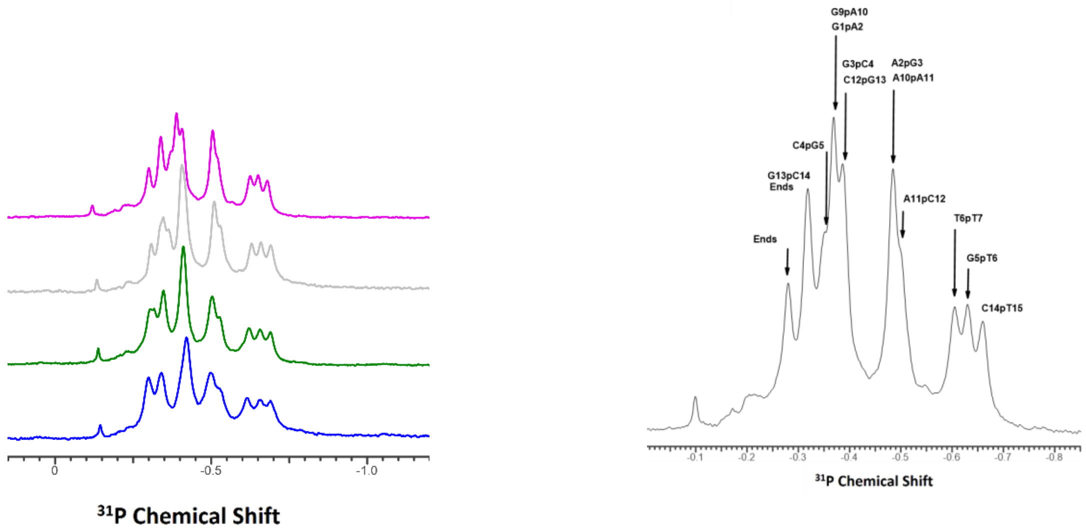

Figure S3. Left) 1D ^{31}P temperature study for the 8mer control DNA. Right) Assignments of 1D ^{31}P temperature study for the 8mer control DNA at 283K. The δP values have been externally referenced to H_3PO_4 at 0.00 ppm via coaxial insert.

<u>**8mer Control %BII Temperature Study**</u>

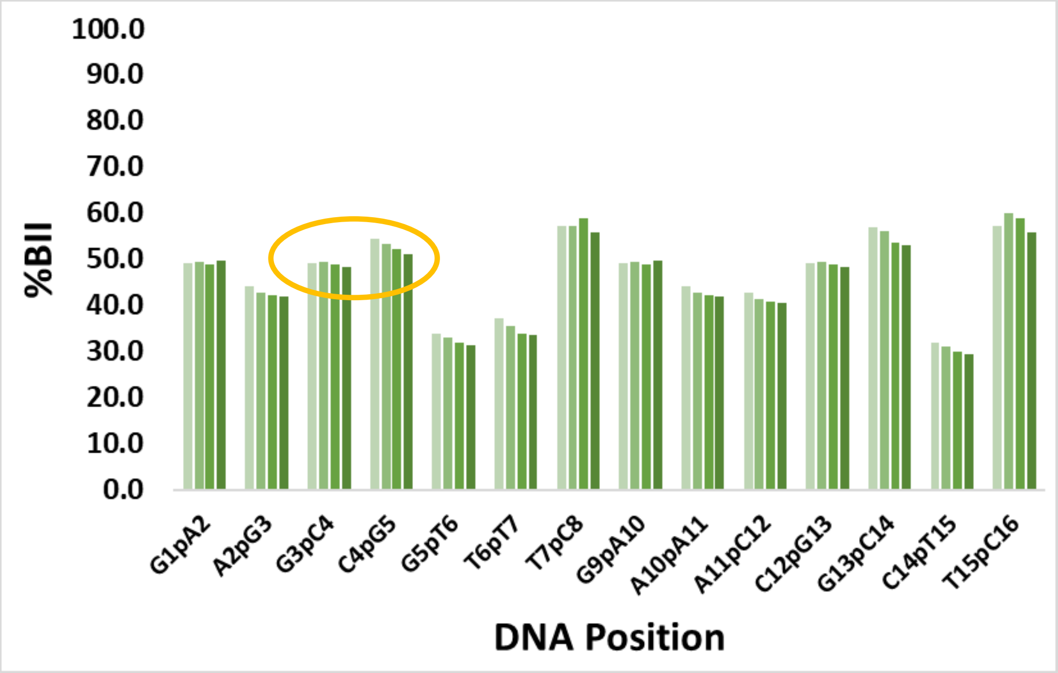

Figure S4. Temperature study of %BII for the 8mer control DNA as a function of sequence position. The temperature values range from 278K (lightest) to 293 (darkest) in 5K steps. The orange oval indicates the phosphate step where the most significant changes are observed in mismatched DNA.

8mer CpG:T DNA

8mer CpG:T ^{1}H Assignments

**8mer CpG:T**	H8/H6	H2/H5/Me	1'	2'	2''	3'	4′
G1	7.91		5.57	2.55	2.78		4.18
A2	8.19		6.12	2.61	2.87		4.44+
G3	7.64		5.91	2.41	2.71		4.42
T4	7.14	1.64	5.48	2.04	2.21	4.82	4.07
G5	7.97		6.10	2.72	2.81		4.44-
T6	7.33	1.43	6.11	2.08	2.58		4.24
T7	7.45	1.70	6.17	2.08	2.58		4.15
C8	7.65	5.81	6.30				4.02
G9	7.89		5.53	2.53	2.76		4.17
A10	8.25		5.94	2.76	2.9		4.43
A11	8.17		6.18	2.61	2.87		4.48
C12	7.25	5.26	5.70	2.04	2.4		4.18
G13	7.78		5.95	2.55	2.68		4.36
C14	7.39	5.33	5.87	1.98	2.44		4.16
T15	7.65	1.67	6.18				4.20
C16	7.65	5.81	6.30				4.02

Table S3. ^{1}H Assignments for **8mer CpG:T** DNA at 283K. The δH values have been referenced to the temperature-dependent HDO signal per Gottlieb et al.

8mer CpG:T ^{1}H Assignments

**8mer CpG:T**	H8/H6	H2/H5/Me	1'	2'	2''	3'	4′
G1	7.90		5.59	2.54	2.79	4.85	4.21
A2	8.15		6.12	2.74	2.96	5.07	4.42
G3	7.60		5.89	2.45	2.74	4.96	4.41
T4	7.12	1.64	5.47	2.07	2.24	4.83	4.06
G5	7.95		6.09	2.73	2.84	5.00	4.42
T6	7.3	1.42	6.10	2.11	2.61	4.87	4.23
T7	7.39	1.66	6.12	2.12	2.55	4.87	4.13
C8	7.49	5.50	6.23	2.25	2.35	4.58	3.96
G9	7.87		5.56	2.57	2.76	4.85	4.21
A10	8.21		5.93	2.77	2.91	5..07	4.41
A11	8.14		6.15	2.64	2.89	5.04	4.47
C12	7.24	5.26	5.69	2.07	2.43	4.79	4.17
G13	7.76		5.94	2.59	2.7	4.91	4.35
C14	7.36	5.31	5.85	2.01	2.47	4.69	4.15
T15	7.48	1.63	6.15	2.18	2.53	4.69	4.19
C16	7.49	5.50	6.26	2.25	2.35	4.58	3.96

Table S4. ^{1}H Assignments for **8mer CpG:T** DNA at 288K. The δH values have been referenced to the temperature-dependent HDO signal per Gottlieb et al.

8mer CpG:T ^{31}P Assignments

DNA position	278K	283K	288K	293K
G1pA2	-0.41	-0.40	-0.40	-0.36
A2pG3	-0.49	-0.49	-0.50	-0.47
G3pT4	-0.78	-0.79	-0.80	-0.78
T4pG5	-0.09	-0.10	-0.11	-0.11
G5pT6	-0.66	-0.66	-0.66	-0.63
T6pT7	-0.60	-0.61	-0.61	-0.59
T7pC8	-0.25	-0.24	-0.25	-0.26
G9pA10	-0.40	-0.40	-0.39	-0.36
A10pA11	-0.45	-0.45	-0.46	-0.45
A11pC12	-0.54	-0.54	-0.55	-0.51
C12pG13	-0.32	-0.32	-0.33	-0.33
G13pC14	-0.36	-0.38	-0.37	-0.35
C14pT15	-0.86	-0.85	-0.84	-0.80
T15pC16	-0.25	-0.24	-0.25	-0.26

Table S5. ^{31}P assignments for **8mer CpG:T** control DNA as a function of temperature. The δP values have been externally referenced to H_3PO_4 at 0.00 ppm via coaxial insert.

8mer CpG:T NOESY Spectra

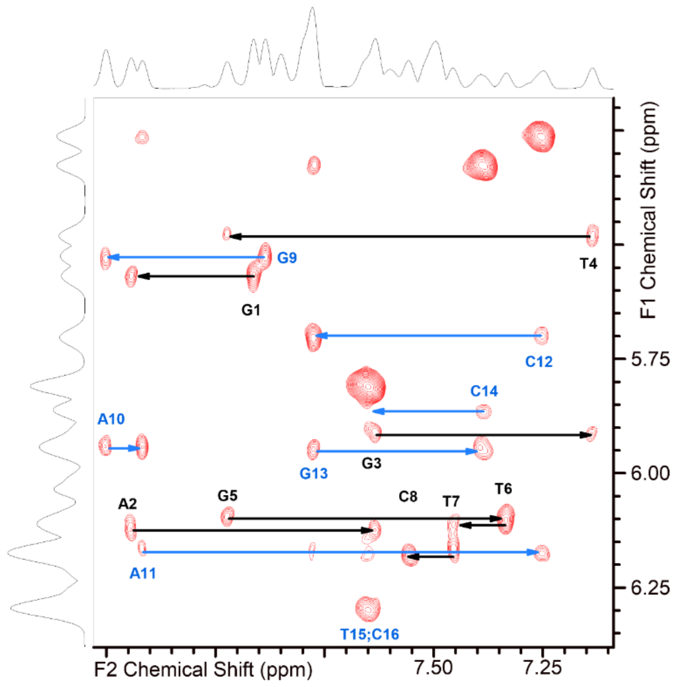

Figure S5. Fingerprint region of the NOESY spectrum of **8mer CpG:T** DNA showing the aromatic–H1′ intranucleotide and sequential connectivities at 283K. All labels are shown above the respective intranucleotide aromatic-1' crosspeak for the indicated nucleotide. The black lines represent the strand containing the mismatched T. The blue lines represent the strand containing the base-paired G. The δH values have been referenced to the temperature-dependent HDO signal per Gottlieb et al.

8mer CpG:T HSQC Spectra

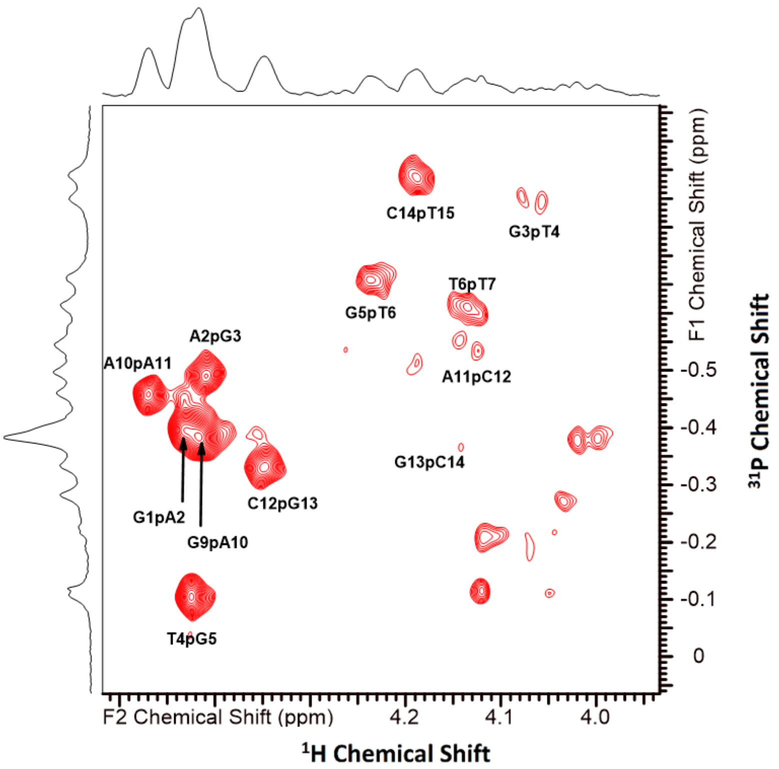

Figure S6. HSQC spectrum of the **8mer CpG:T** sequence at 288K indicating the H4' to ^{31}P crosspeaks with the internucleotide phosphate indicated. Note the F2 axis (^{31}P) has been externally referenced to H_3PO_4 at 0.00 ppm via coaxial insert. The F1 axis represents the 4' proton for the 5' nucleotide of the dinucleotide pair.

8mer CpG:T 1D ^{31}P Temperature Study

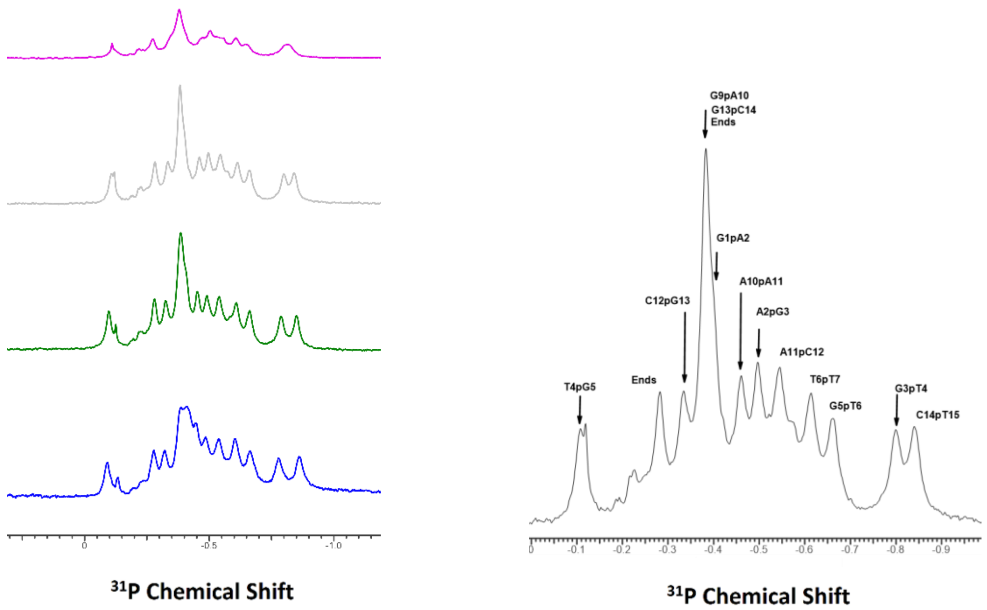

Figure S7. Left) 1D ^{31}P temperature study for the **8mer CpG:T** DNA. Right) Assignments of 1D ^{31}P temperature study for the 8mer control DNA at 288K. The δP values have been externally referenced to H_3PO_4 at 0.00 ppm via coaxial insert.

8mer CpG:T %BII Temperature Study

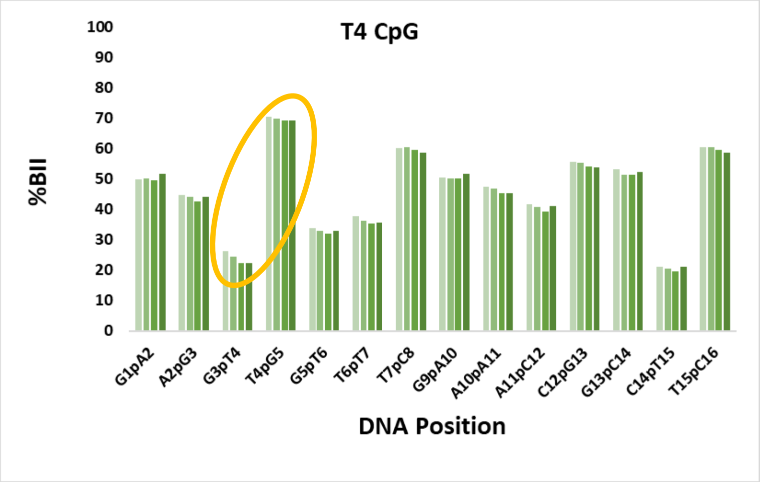

Figure S8. Temperature study of %BII for the **8mer CpG:T** DNA as a function of sequence position. The temperature values range from 278K (lightest) to 293 (darkest) in 5K steps. The orange oval indicates the phosphate step where the most significant changes are observed relative to canonical DNA.

8mer TpG:T DNA

8mer TpG:T ^{1}H Assignments

**8mer TpG:T**	H8/H6	H2/H5/Me	1'	2'	2''	3'	4′
G1	7.90		5.59	2.54	2.79	4.85	4.21+
A2	8.15		6.12	2.74	2.96	5.07	4.42+
G3	7.60		5.89	2.45	2.74	4.96	4.41
T4	7.12	1.64	5.47	2.07	2.24	4.83	4.06
A5	7.95		6.09	2.73	2.84	5.00	4.42
T6	7.30	1.42	6.10	2.11	2.61	4.87	4.23
T7	7.39	1.66	6.12	2.12	2.55	4.87	4.13
C8	7.49	5.50	6.23	2.25	2.35	4.58	3.96
G9	7.87		5.56	2.57	2.76	4.85	4.21
A10	8.21		5.93	2.77	2.91	5.07	4.41+
A11	8.14		6.15	2.64	2.89	5.04	4.47
T12	7.24	5.26	5.69	2.07	2.43	4.79	4.17
G13	7.76		5.94	2.59	2.7	4.91	4.35
C14	7.36	5.31	5.85	2.01	2.47	4.69	4.15
T15	7.48	1.63	6.15	2.18	2.53	4.69	4.19
C16	7.49	5.50	6.26	2.25	2.35	4.58	3.96

Table S6. ^{1}H Assignments for **8mer TpG:T** DNA at 288K. The δH values have been referenced to the temperature-dependent HDO signal per Gottlieb et al.

8mer TpG:T ^{31}P Assignments

DNA position	278K	283K	288K	293K
G1pA2	-0.48	-0.47	-0.44	
A2pG3	-0.49	-0.50	-0.49	
G3pT4	-0.77	-0.78	-0.77	-0.73
T4pA5	-0.30	-0.32	-0.32	
A5pT6	-0.70	-0.69	-0.67	-0.62
T6pT7	-0.65	-0.65	-0.63	-0.60
T7pC8	-0.27	-0.27	-0.27	
G9pA10	-0.48	-0.47	-0.44	
A10pA11	-0.41	-0.43	-0.42	
A11pT12	-0.60	-0.61	-0.60	-0.57
T12pG13	-0.50	-0.51	-0.50	-0.51
G13pC14	-0.49	-0.48	-0.48	
C14pT15	-0.85	-0.85	-0.82	
T15pC16	-0.27	-0.27	-0.27	

Table S7. ^{31}P assignments for **8mer TpG:T** control DNA as a function of temperature. The δP values have been externally referenced to H_3PO_4 at 0.00 ppm via coaxial insert.

8mer TpG:T NOESY Spectra

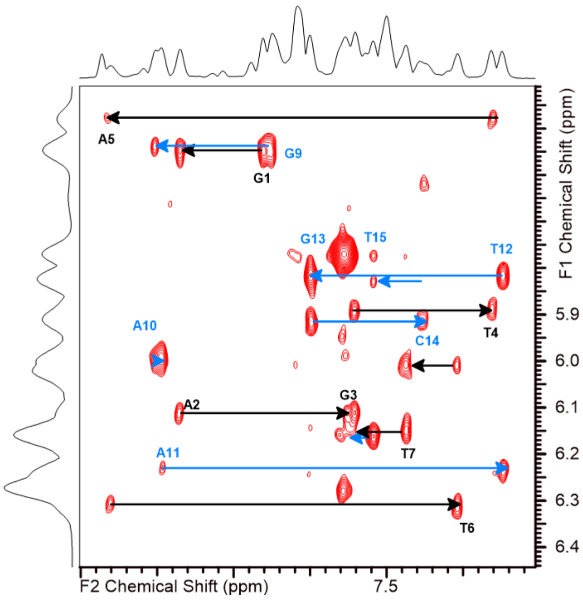

Figure S9. Fingerprint region of the NOESY spectrum of **8mer TpG:T** DNA showing the aromatic−H1' intranucleotide and sequential connectivities at 283K. All labels are shown above the respective intranucleotide aromatic-1' crosspeak for the indicated nucleotide. The black lines represent the strand containing the mismatched T. The blue lines represent the strand containing the base-paired G. The δH values have been referenced to the temperature-dependent HDO signal per Gottlieb et al.

8mer TpG:T HSQC Spectra

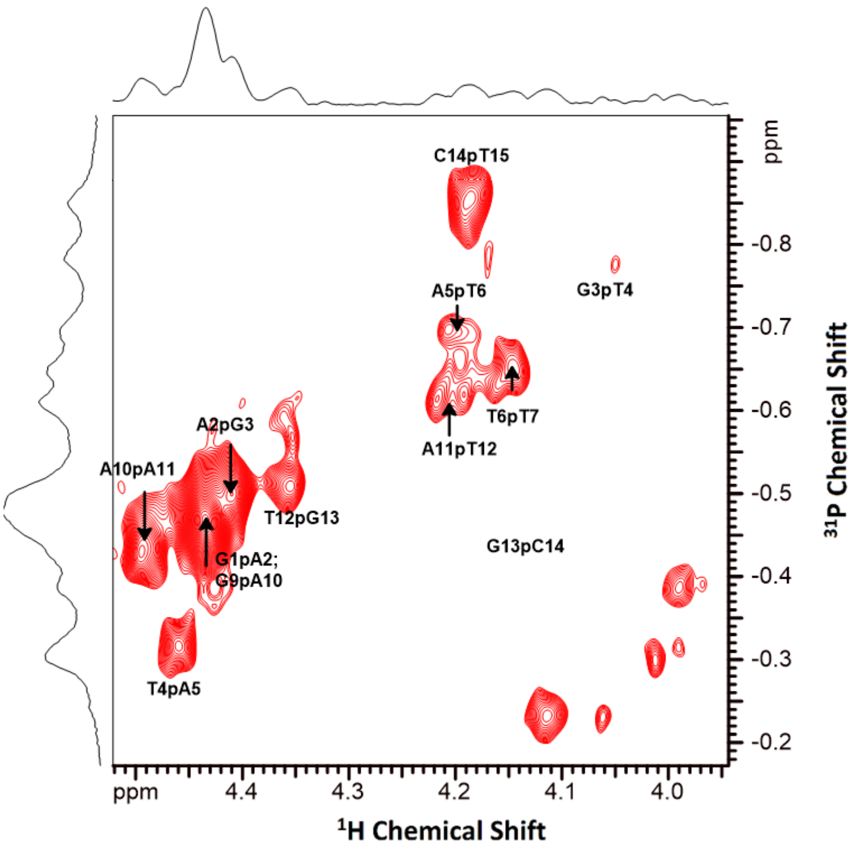

Figure S10. HSQC spectrum of the **8mer TpG:T** sequence at 288K indicating the H4' to ^{31}P crosspeaks with the internucleotide phosphate indicated. Note the F2 axis (^{31}P) has been externally referenced to H_3PO_4 at 0.00 ppm via coaxial insert. The F1 axis represents the 4' proton for the 5' nucleotide of the dinucleotide pair.

<u>8mer TpG:T %BII Temperature Study</u>

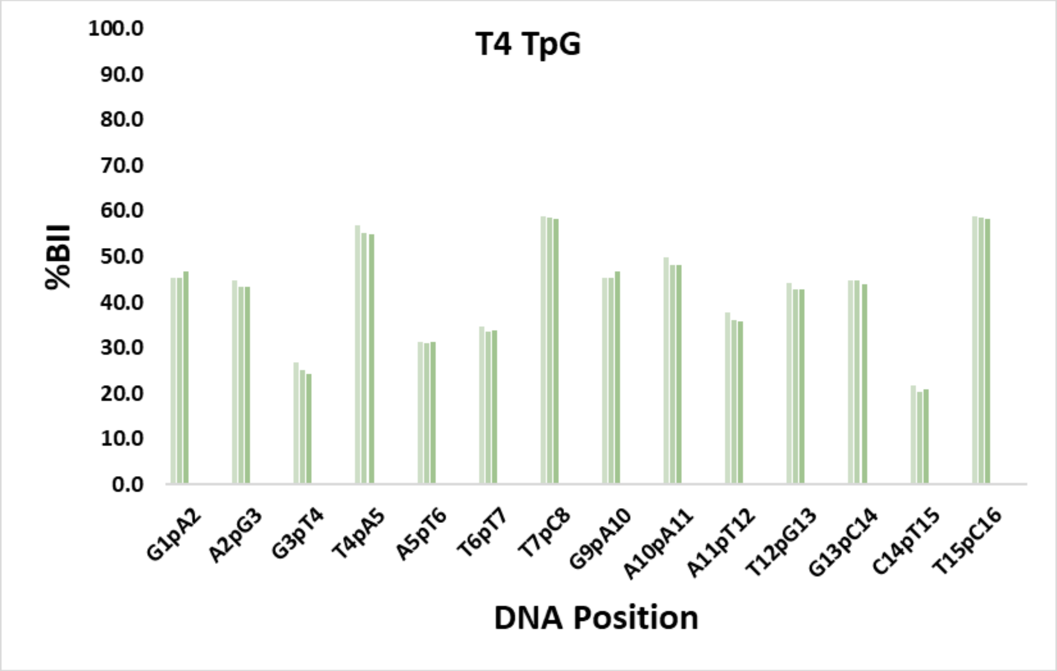

Figure S11. Temperature study of %BII for the **<u>8mer TpG:T</u>** DNA as a function of sequence position. The temperature values range from 278K (lightest) to 288K (darkest) in 5K steps. Note that unlike the other sequences, these data only go to 288K as the quality and accuracy of the data at higher temperatures was suspect for several positions in this sequence.

8mer GpG:T DNA

8mer GpG:T ^{1}H Assignments

**8mer GpG:T**	H8/H6	H2/H5/Me	1'	2'	2''	3'	4′
G1	7.93		5.60	2.58	2.77	4.87	4.21
A2	8.22		6.14	2.79	2.97	5.09	4.47
G3	7.66		5.95	2.48	2.77	4.96	4.45
T4	7.17	1.60	5.79	2.06	2.38	4.83	4.13
C5	7.69	5.60	6.09	2.26	2.51	4.87	4.27
T6	7.51	1.68	6.09	2.20	2.63	4.91	4.22-
T7	7.49	1.75	6.17	2.20	2.58	4.91	4.17+
C8	7.65		6.30	2.30	2.3	4.61	4.03
G9	7.91		5.52	2.53	2.72	4.85	4.18
A10	8.26		5.88	2.80	2.91	5.08-	4.43
A11	8.13		6.05	2.68	2.91	5.08+	4.48
G12	7.61		5.74	2.48	2.67	4.94	4.38
G13	7.54		5.97	2.62	2.69	4.83	4.42
C14	7.37		5.84	2.05	2.51	4.70	4.17-
T15	7.59	1.66	6.19	2.20	2.58	4.91	4.22+
C16	7.65		6.30	2.30	2.30	4.61	4.03

Table S8. ^{1}H Assignments for **8mer GpG:T** DNA at 288K. The δH values have been referenced to the temperature-dependent HDO signal per Gottlieb et al.

8mer GpG:T ^{31}P Assignments

DNA position	278K	283K	288K	293K
G1pA2	-0.40	-0.41	-0.40	-0.40
A2pG3	-0.43	-0.43	-0.43	-0.43
G3pT4	-0.69	-0.70	-0.70	-0.70
T4pC5	-0.40	-0.41	-0.40	-0.40
C5pT6	-0.53	-0.53	-0.52	-0.52
T6pT7	-0.62	-0.62	-0.61	-0.60
T7pC8	-0.25	-0.25	-0.24	-0.24
G9pA10	-0.37	-0.38	-0.36	-0.36
A10pA11	-0.40	-0.41	-0.40	-0.40
A11pG12	-0.30	-0.30	-0.30	-0.32
G12pG13	-0.30	-0.30	-0.30	-0.32
G13pC14	-0.51	-0.50	-0.49	-0.49
C14pT15	-0.89	-0.88	-0.86	-0.84
T15pC16	-0.25	-0.25	-0.24	-0.24

Table S9. ^{31}P assignments for **8mer GpG:T** control DNA as a function of temperature. The δP values have been externally referenced to H_3PO_4 at 0.00 ppm via coaxial insert.

8mer GpG:T NOESY Spectra

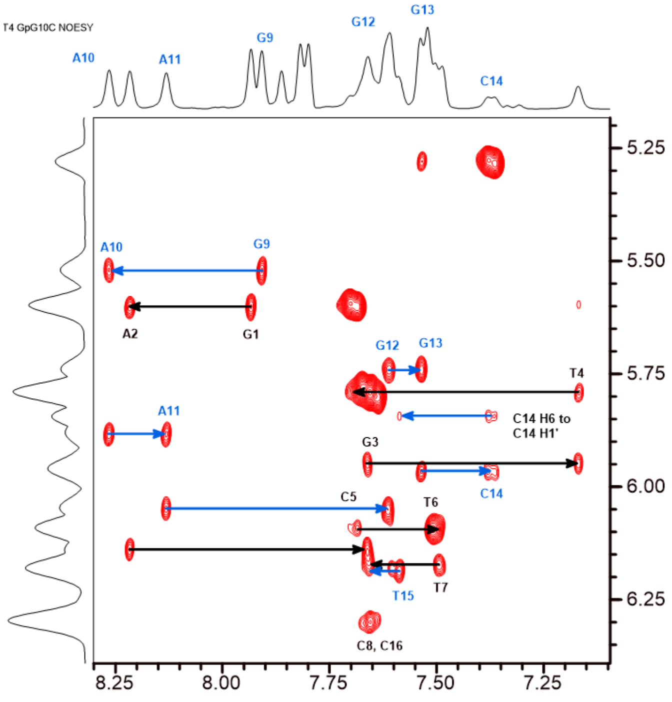

Figure S12. Fingerprint region of the NOESY spectrum of **8mer GpG:T** DNA showing the aromatic−H1′ intranucleotide and sequential connectivities at 283K. All labels are shown above the respective intranucleotide aromatic-1' crosspeak for the indicated nucleotide. The black lines represent the strand containing the mismatched T. The blue lines represent the strand containing the base-paired G. The δH values have been referenced to the temperature-dependent HDO signal per Gottlieb et al.

8mer GpG:T HSQC Spectra

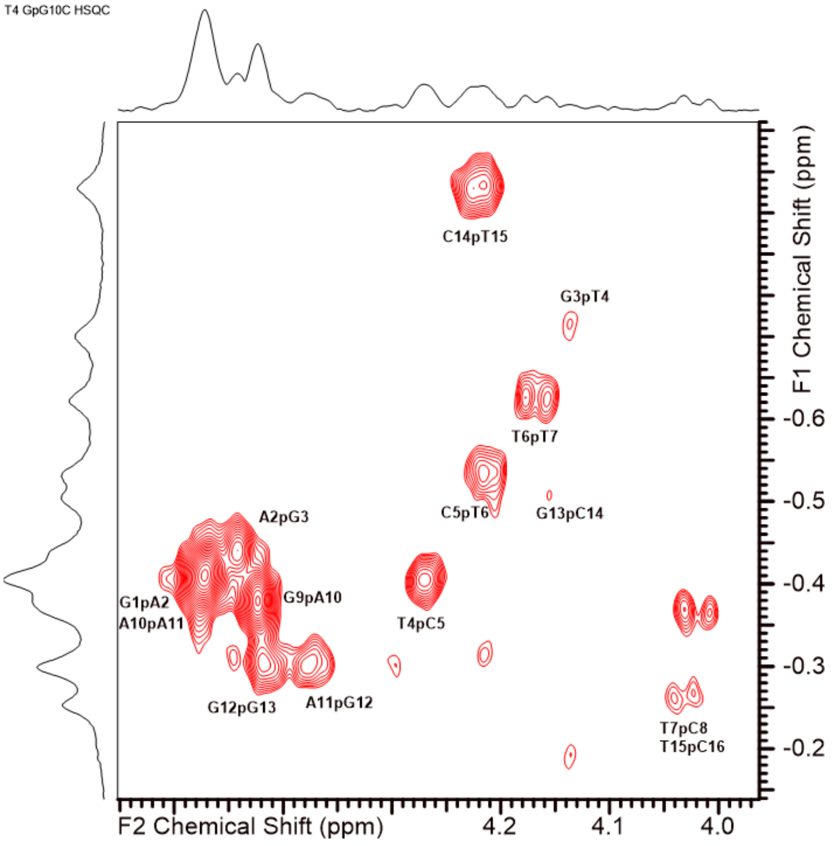

Figure S13. HSQC spectrum of the **8mer GpG:T** sequence at 283K indicating the H4' to ^{31}P crosspeaks with the internucleotide phosphate indicated. Note the F2 axis (^{31}P) has been externally referenced to H_3PO_4 at 0.00 ppm via coaxial insert. The F1 axis represents the 4' proton for the 5' nucleotide of the dinucleotide pair.

8mer GpG:T 1D ^{31}P Temperature Study

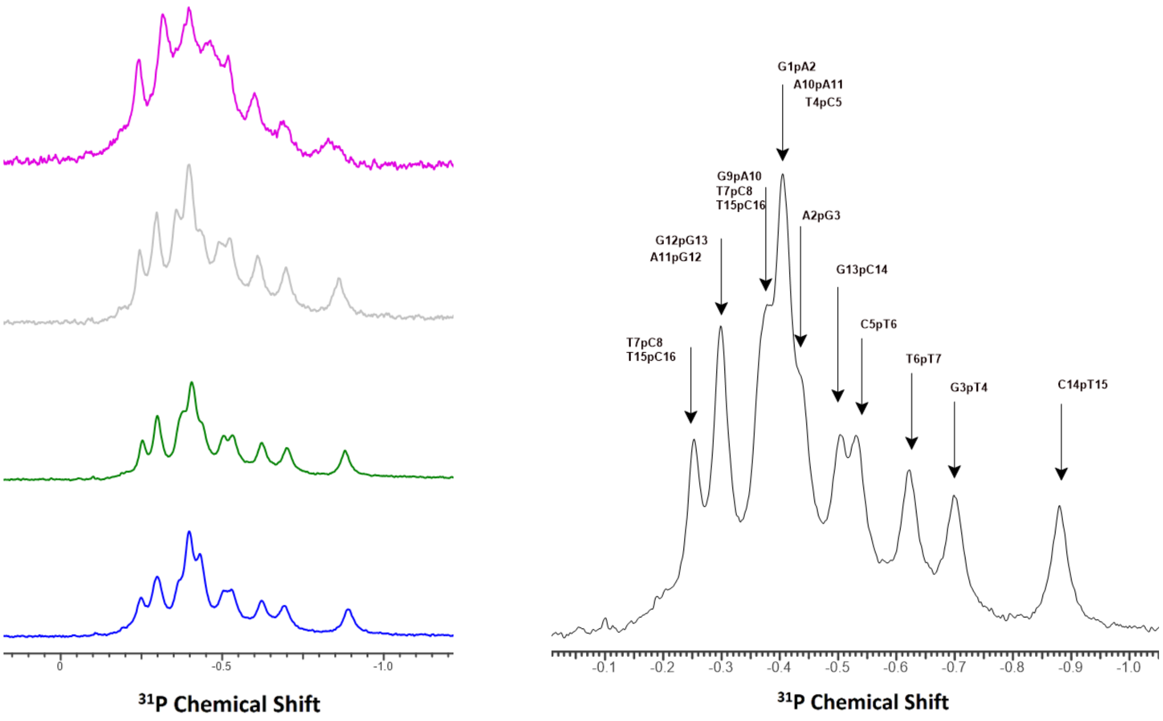

Figure S14. Left) 1D ^{31}P temperature study for the **<u>8mer GpG:T</u>** DNA. Right) Assignments of 1D ^{31}P temperature study for the 8mer control DNA at 288K. The δP values have been externally referenced to H_3PO_4 at 0.00 ppm via coaxial insert.

8mer GpG:T %BII Temperature Study

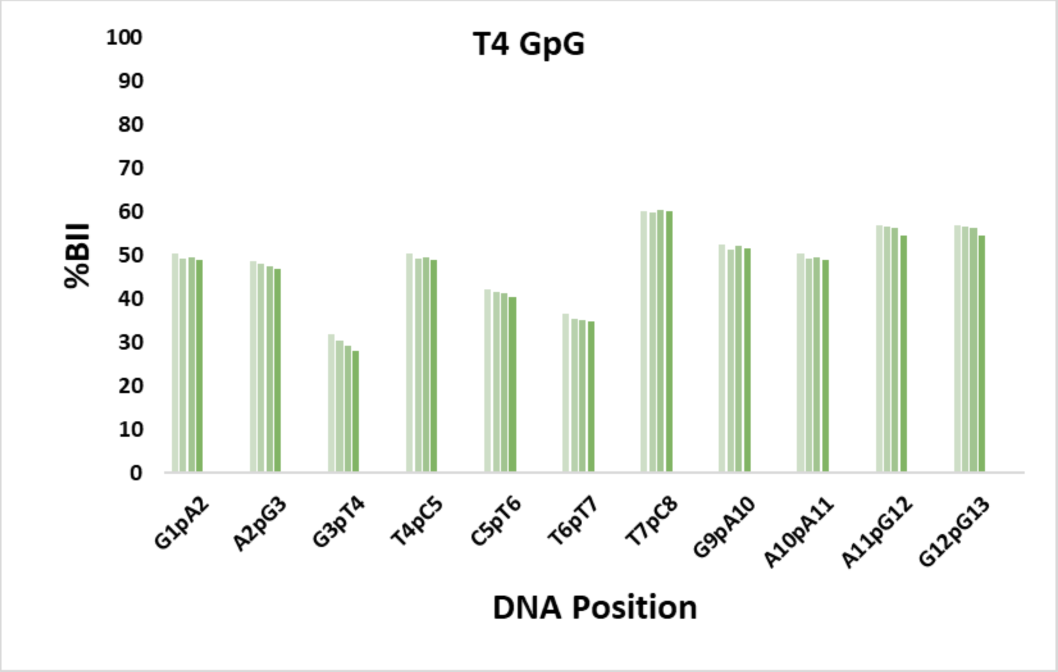

Figure S15. Temperature study of %BII for the **8mer GpG:T** DNA as a function of sequence position. The temperature values range from 278K (lightest) to 293 (darkest) in 5K steps.

8mer ApG:T DNA

8mer ApG:T ^{1}H Assignments

**8mer ApG:T**	H8/H6	H2/H5/Me	1'	2'	2''	3'	4′
G1	7.91		5.55	2.54	2.75	4.85	4.20
A2	8.15		6.08	2.70	2.91	5.06	4.43
G3	7.63		5.86	2.44	2.68	4.94	4.42
T4	7.17	1.59	5.72	1.89	2.39	4.76	4.09
T5	7.57	1.51	6.12	2.31	2.54	4.9	4.26
T6	7.53	1.62	6.1	2.09-2.17	2.52-2.62	4.87	4.19
T7	7.43	1.66	6.1	2.09	2.52-2.62	4.87-4.91	4.15
C8						4.57	
G9	7.87		5.49	2.48	2.69	4.82	4.18
A10	8.20		5.76	2.72	2.82	5.05	4.38
A11	8.13		5.88	2.65	2.87	5.07	4.45
A12	8.05		5.98	2.57	2.77	5.00	4.43
G13	7.43		5.79	2.47	2.59	4.80	4.36
C14	7.3		5.76	1.97	2.42	4.64	4.13
T15	7.52	1.55	6.12	2.09-2.17	2.52-2.62	4.86	4.19
C16						4.57	

Table S10. ^{1}H Assignments for **8mer ApG:T** DNA at 278K. The δH values have been referenced to the temperature-dependent HDO signal per Gottlieb et al. Note that some protons were unable to be assigned unambiguously.

8mer ApG:T ^{31}P Assignments

DNA position	278K	283K	288K	293K
G1pA2	-0.45	-0.45	-0.48	-0.49
A2pG3	-0.45	-0.45	-0.48	-0.49
G3pT4	-0.71	-0.72	-0.70	-0.66
T4pT5	-0.75	-0.75	-0.73	-0.66
T5pT6	-0.62	-0.58	-0.57	-0.55
T6pT7	-0.59	-0.58	-0.57	-0.55
T7pC8	-0.24	-0.24	-0.24	-0.22
G9pA10	-0.44	-0.45	-0.44	-0.42
A10pA11	-0.42	-0.42	-0.44	-0.42
A11pA12	-0.45	-0.45	-0.36	-0.42
A12pG13	-0.31	-0.32	-0.36	-0.4
G13pC14	-0.51	-0.58	-0.57	-0.54
C14pT15	-0.90	-0.9	-0.86	-0.78
T15pC16	-0.24	-0.24	-0.24	-0.22

Table S11. ^{31}P assignments for **8mer ApG:T** control DNA as a function of temperature. The δP values have been externally referenced to H_3PO_4 at 0.00 ppm via coaxial insert.

8mer ApG:T NOESY Spectra

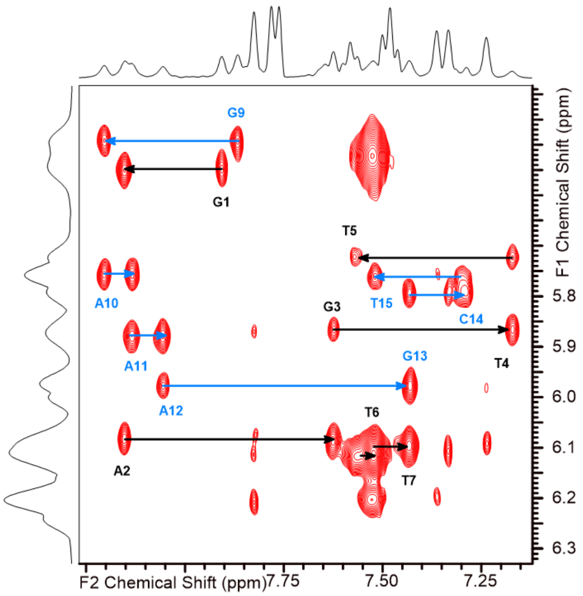

Figure S16. Fingerprint region of the NOESY spectrum of **8mer ApG:T** DNA showing the aromatic−H1' intranucleotide and sequential connectivities at 283K. All labels are shown above the respective intranucleotide aromatic-1' crosspeak for the indicated nucleotide. The black lines represent the strand containing the mismatched T. The blue lines represent the strand containing the base-paired G. The δH values have been referenced to the temperature-dependent HDO signal per Gottlieb et al.

8mer ApG:T HSQC Spectra

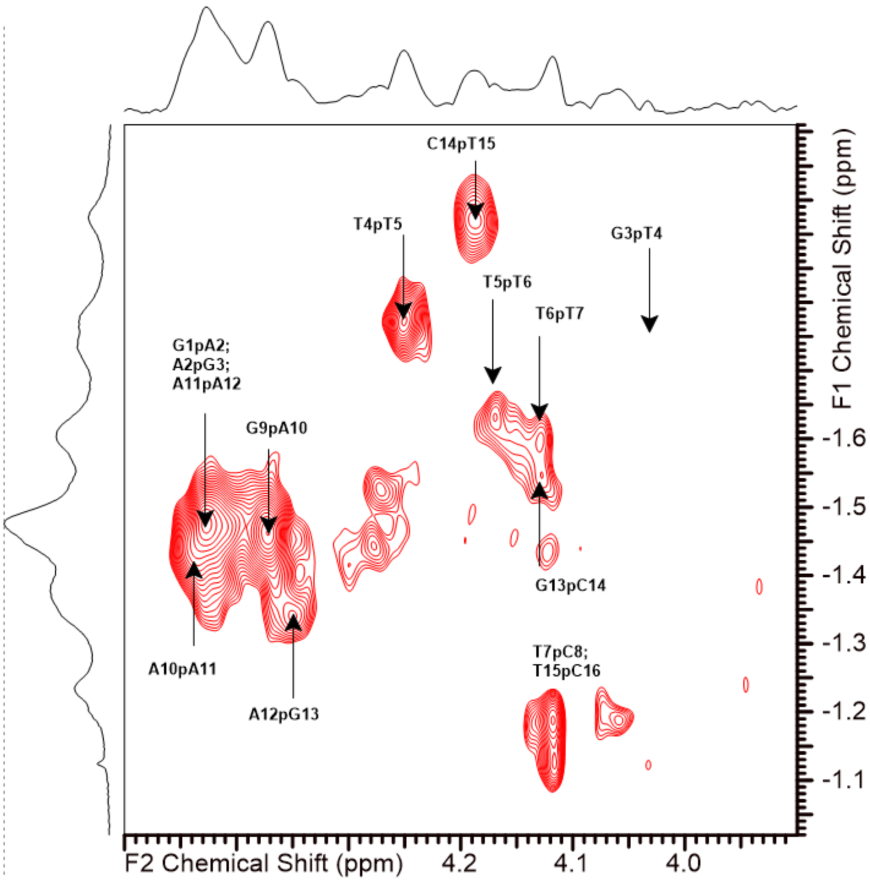

Figure S17. HSQC spectrum of the **8mer ApG:T** sequence at 288K indicating the H4' to ^{31}P crosspeaks with the internucleotide phosphate indicated. Note the F2 axis (^{31}P) has been externally referenced to H_3PO_4 at 0.00 ppm via coaxial insert. The F1 axis represents the 4' proton for the 5' nucleotide of the dinucleotide pair.

8mer ApG:T 1D ^{31}P Temperature Study

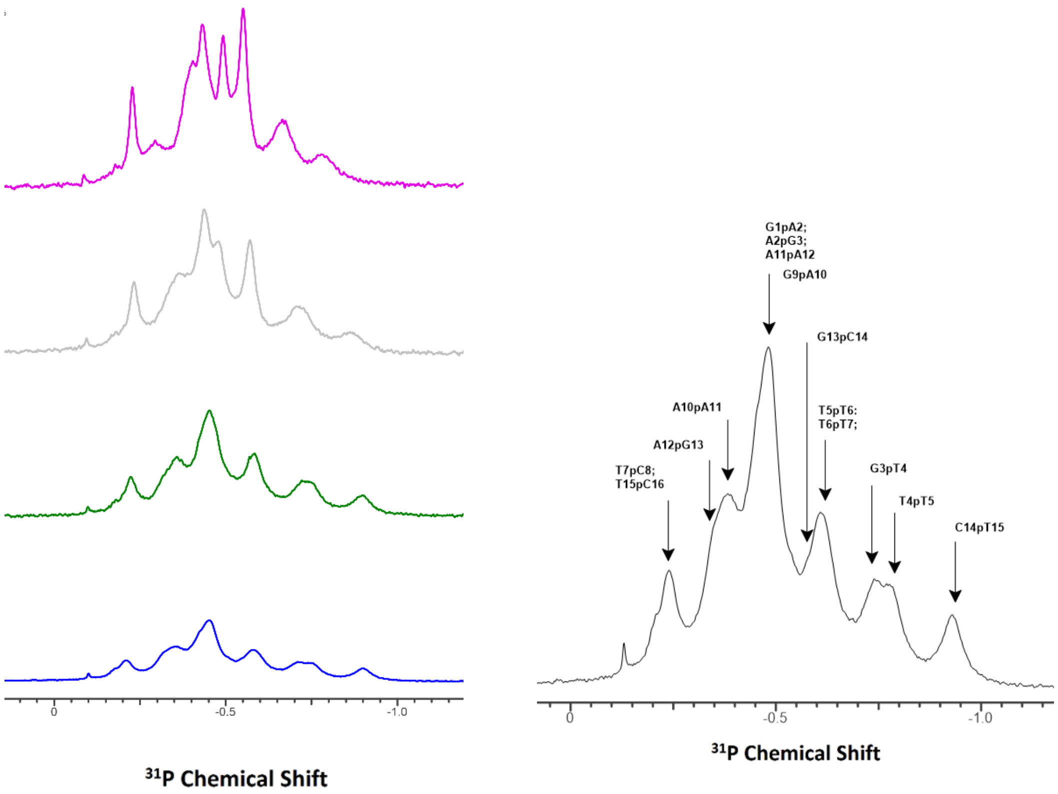

Figure S18. Left) 1D ^{31}P temperature study for the **8mer ApG:T** DNA. Right) Assignments of 1D ^{31}P temperature study for the 8mer control DNA at 288K. The δP values have been externally referenced to H_3PO_4 at 0.00 ppm via coaxial insert.

8mer ApG:T %BII Temperature Study

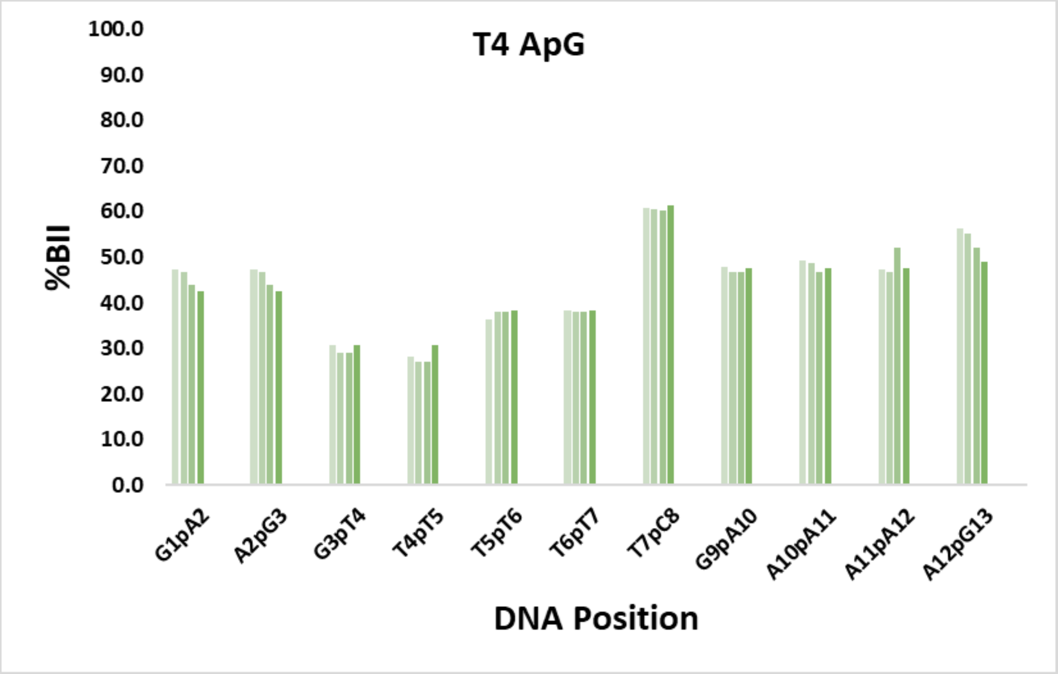

Figure S19. Temperature study of %BII for the **8mer ApG:T** DNA as a function of sequence position. The temperature values range from 278K (lightest) to 293K (darkest) in 5K steps.

Adapted with permission from 1kkw PDB structure (reference 60). Copyright 2002 Elsevier. CpG:T dinucleotide context, highest enzyme activity

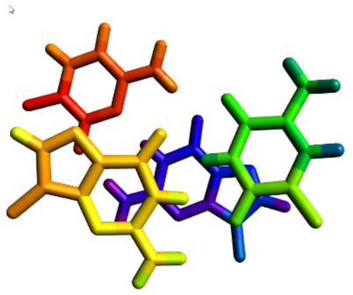

Adapted with permission from 1bjd PDB structure (reference 59). Copyright 1998 Oxford University Press. TpG:T dinucleotide context; second highest enzyme activity

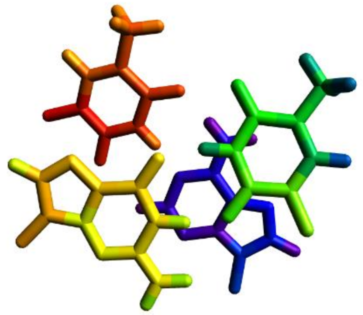

Adapted with permission from Pfaff et al. structure (reference 61). Copyright 2008 American Chemical Society. ApG:T, lowest affinity

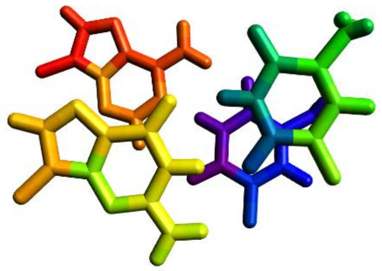

Figure S20. Comparison of the 5'-TpX-3':5'-YpG-3' dinucleotides from the structures in references 59 – 61, to qualitatively view the base stacking. Using Avogadro (an open-source molecular builder and visualization tool. Version 1.2.0. http://avogadro.cc/), we have color-coded the relevant bases to ease viewing: green = mismatched T; yellow = base-paired G; blue = 3' stacking neighbor to T; orange = 5' stacking neighbor to G
